# A Model Guided Approach to Evoke Homogeneous Behavior During Temporal Reward and Loss Discounting

**DOI:** 10.3389/fpsyt.2022.846119

**Published:** 2022-06-21

**Authors:** Janine Thome, Mathieu Pinger, Patrick Halli, Daniel Durstewitz, Wolfgang H. Sommer, Peter Kirsch, Georgia Koppe

**Affiliations:** ^1^Department of Theoretical Neuroscience, Central Institute of Mental Health, Medical Faculty Mannheim, Heidelberg University, Mannheim, Germany; ^2^Department of Psychiatry and Psychotherapy, Central Institute of Mental Health, Medical Faculty Mannheim, Heidelberg University, Mannheim, Germany; ^3^Department of Clinical Psychology, Central Institute of Mental Health, Medical Faculty Mannheim, Heidelberg University, Mannheim, Germany; ^4^Institute for Psychopharmacology, Central Institute of Mental Health, Medical Faculty Mannheim, Heidelberg University, Mannheim, Germany; ^5^Institute of Psychology, Heidelberg University, Heidelberg, Germany

**Keywords:** temporal discounting, loss discounting, design optimization, reward discounting, computational modeling, computational psychiatry

## Abstract

**Background:**

The tendency to devaluate future options as a function of time, known as delay discounting, is associated with various factors such as psychiatric illness and personality. Under identical experimental conditions, individuals may therefore strongly differ in the degree to which they discount future options. In delay discounting tasks, this inter-individual variability inevitably results in an unequal number of discounted trials per subject, generating difficulties in linking delay discounting to psychophysiological and neural correlates. Many studies have therefore focused on assessing delay discounting adaptively. Here, we extend these approaches by developing an adaptive paradigm which aims at inducing more comparable and homogeneous discounting frequencies across participants on a dimensional scale.

**Method:**

The proposed approach probabilistically links a (common) discounting function to behavior to obtain a probabilistic model, and then exploits the model to obtain a formal condition which defines how to construe experimental trials so as to induce any desired discounting probability. We first infer subject-level models on behavior on a non-adaptive delay discounting task and then use these models to generate adaptive trials designed to evoke graded relative discounting frequencies of 0.3, 0.5, and 0.7 in each participant. We further compare and evaluate common models in the field through out-of-sample prediction error estimates, to iteratively improve the trial-generating model and paradigm.

**Results:**

The developed paradigm successfully increases discounting behavior during both reward and loss discounting. Moreover, it evokes graded relative choice frequencies in line with model-based expectations (i.e., 0.3, 0.5, and 0.7) suggesting that we can successfully homogenize behavior. Our model comparison analyses indicate that hyperboloid models are superior in predicting unseen discounting behavior to more conventional hyperbolic and exponential models. We report out-of-sample error estimates as well as commonalities and differences between reward and loss discounting, demonstrating for instance lower discounting rates, as well as differences in delay perception in loss discounting.

**Conclusion:**

The present work proposes a model-based framework to evoke graded responses linked to cognitive function at a single subject level. Such a framework may be used in the future to measure cognitive functions on a dimensional rather than dichotomous scale.

## Introduction

Evaluating and deciding between alternative outcomes available at different points in time forms one critical aspect of human decision making (1). Outcomes which lie in the distant future are typically devaluated in this context, a phenomenon widely known as temporal or delay discounting (1–3).

Devaluation of future outcomes is *per se* a rational choice strategy as time comes at a cost (2, 4–6), however, some forms of temporal discounting as well as overly steep discounting may result in non-optimal and potentially harmful choices. For instance, it has been argued that steeper (hyperbolic) delay discounting may explain why individuals choose a cigarette now over a long-term healthy life, that is, why they prefer a smaller immediate over a delayed larger reward [(7–9); for overviews see (10, 11)].

In line with this argument, individuals with impulsive disorders and addiction show steeper discounting of future rewards such as monetary gains [for overviews see (12–15)]. Moreover, steeper discounting does not only differentiate between addiction disorders and healthy individuals, but it also predicts entry into drug use as well as therapy outcome (16, 17), and has accordingly been described as a behavioral biomarker of addiction and its treatment (18). Alterations in the discounting of future monetary losses are less well investigated which is surprising given that “continued use despite aversive consequences” is a primary symptom of addiction (19). In any case, understanding the neurobiological mechanisms underlying both temporal reward and loss discounting is therefore of particular clinical concern in the addiction field.

The common way to assess temporal (reward) discounting is the intertemporal choice task (ICT), in which an individual is presented with a series of trials and asked to choose between an immediate smaller vs. a delayed larger reward, or between two options delayed at different time points [(20); for overviews see (3, 21, 22)]. Immediate choices are then taken as an indicator of temporal discounting.

Individuals strongly vary in their tendency to discount due to various factors such as psychiatric illness, but also gender, or personality traits [(12, 23, 24); for overviews see (25, 26)]. In the ICT, this inter-individual variability results in an unequal number of discounted trials per participant, generating difficulties in linking the temporal discount process to psychophysiological and neural correlates. For instance, investigating the underlying neurobiological substrates by comparing differences between immediate and delayed choices may fall short of statistical power given highly unbalanced trial types and the high variability in discounting strength across individuals [e.g., (27–32); for an overview see (21)]. At times, participants even have to be excluded from analyses due to not discounting at all [e.g., (31, 33–45)].

To remedy this problem, delay discounting has often been investigated within adaptive experimental designs. Earlier studies have focused on applying titration procedures [originally introduced by Oldfield (46)] where reward or delay schedules are adjusted on a trial-by-trial basis depending on the participant's choice history in order to find the points at which immediate and delayed choices are displayed with equal probability, the so called ‘indifference points' (since at these points the participant is indifferent toward either choice [e.g., (7, 9, 20, 27–29, 37, 47–51)]).

More recently, several behavioral model-based approaches have been proposed which aim at adapting the ICT trials to the individual so as to elicit (more) comparable levels of discounting and assess discounting more efficiently (31, 32, 36, 43, 52–56). Ordinarily these approaches make the assumption that the devaluation of future outcomes follows a hyperbolic curve such that the perceived outcome values monotonically decrease with increasing delay. Consequently, there are unique points on this curve where the perceived value of a delayed larger outcome and an immediate smaller outcome intersect, that is, where immediate and delayed outcome values are equal, corresponding to the individual's indifference points.

While some of these approaches infer discount parameters with a remarkably low number of trials [e.g., (36, 57)], their primary goal lies in the efficient inference of subject-wise discounting parameters and/or in determining inter-individual indifference points. The latter are then used to contrast neural activation toward “hard” as compared to “easy” adaptive trials (i.e., trials close to vs. far from the indifference point) (58), or to compare immediate and delayed responses with comparable frequency at the indifference points. However, since discounting is described by a continuous monotone function, it may in principle be interesting to study the neural response not only at the indifference point of a subject at which we expect a 0.5 discounting probability [see also (59, 60)]. By parametrically mapping the individual discounting curves onto behavioral probabilities comparable across subjects, we may construe experimental trials which allow us to examine discounting behavior and its neural correlates dimensionally. At the same time, by constructing customized trials for a given discounting probability, we may create more homogeneous experimental conditions on the behavioral discounting continuum, and thereby increase statistical power needed to compare discounting behavior at different levels (e.g., low, medium, and high).

Another caveat of the model-based approaches is that they almost exclusively rely on the hyperbolic discounting model (with one exception (53)), and thus depend on the implicit assumption of this model being ‘true', or at least on it accounting for a substantial proportion of intra-individual variability. However, studies primarily focusing on comparing the goodness-of-fit of different discounting functions have suggested that this may not be the case [(53); see also (38, 49, 61–63)], such that multiple alternatives to the hyperbolic model have been proposed [(53); see also (5, 27, 64–69)]. Adaptive design procedures may therefore benefit from taking into account model comparison results.

Here, we propose a generic framework which generates individualized experimental trials based on a proposed model, and subsequently evaluates a variety of models in order to create an adaptive experimental (discounting) paradigm (following the pre-registered protocol: https://doi.org/10.17605/OSF.IO/PMWXB). In contrast to previous studies, our framework provides a formal condition to generate trials which are expected to elicit graded discounting probabilities on a dimensional scale. Models are selected based on out-of-sample estimates of the prediction error (70), such that we can report how well the tested models perform at predicting unseen data. We also extend the paradigm to loss discounting. The proposed framework may be transferred to generate experimental paradigms tailored to the assessment of other cognitive or emotional functions.

## Materials and Methods

### Study Design

**Figure 1:**
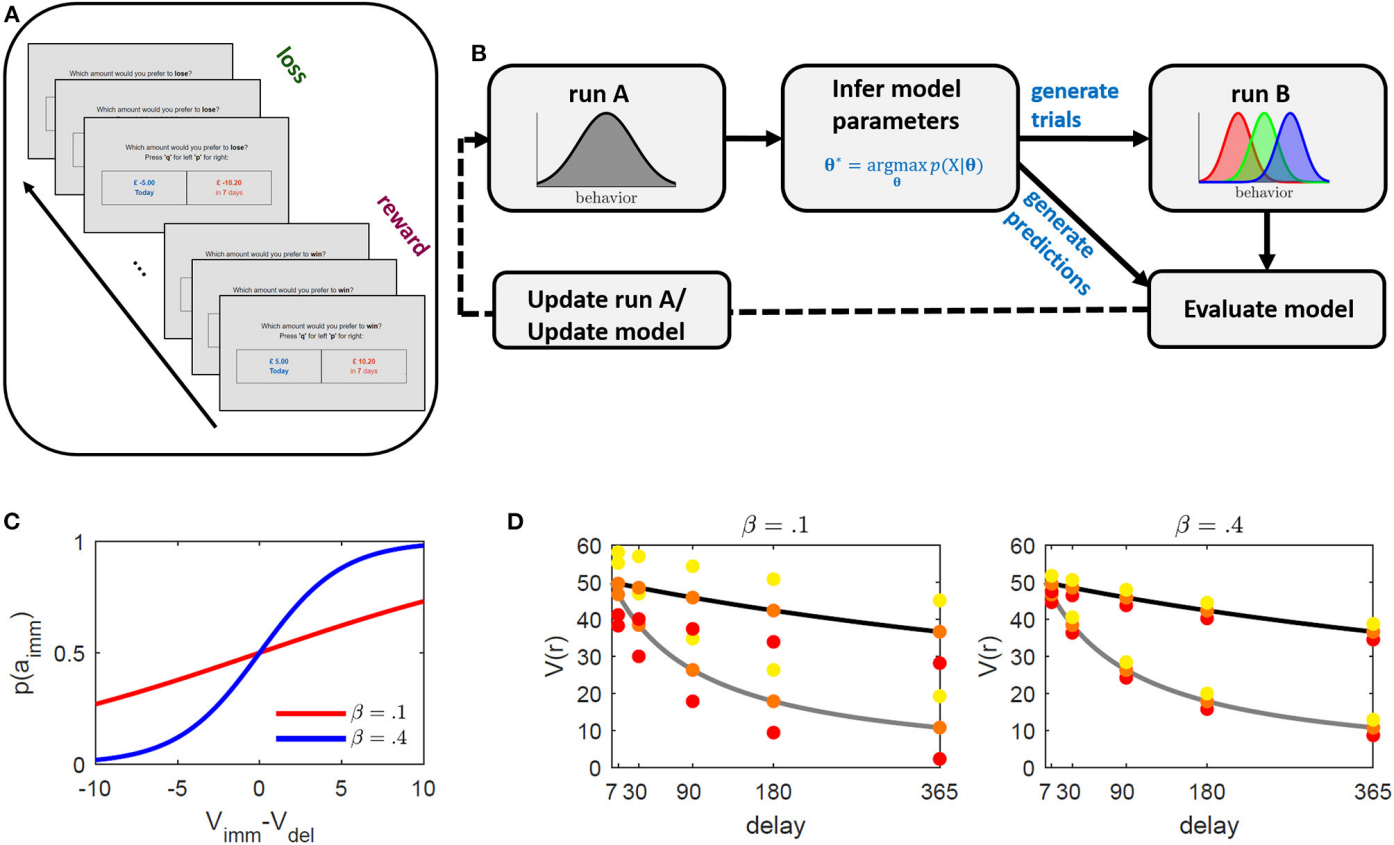
Illustration of the intertemporal choice task (ICT) and proposed paradigm adaptation framework. **(A)** Schematic illustration of the ICT. Subjects were faced with a series of binary choice trials between an immediate and a delayed outcome. The absolute value of the delayed outcome was always higher. Reward and loss trials were presented within alternating blocks of 40 trials each. **(B)** Paradigm development framework. Subjects perform an ICT with equal trials across subjects (run A). The task is used to infer subject-level parameters based on a proposed underlying behavioral model. These parameters are used to generate individualized trials designed to elicit relative immediate choice frequencies of 0.3, 0.5, and 0.7 of run B (schematically displayed as red, green, and blue, respectively), and to generate behavioral predictions along with other common discounting models. By comparing observed and predicted behavior in run B, the task underlying model is either updated, or trials of run A are optimized to improve parameter inference. The procedure may be repeated until no further improvement is observed. **(C, D)** Illustration of method's operating principle. **(C)** Immediate choice probability *p*(a_imm_) (cf. Equation 2) as a function of the difference between immediate (*V*_imm_) and delayed (*V*_del_) value for β = 0.1 (red), and β = 0.4 (blue). The indifference point where *V*_imm_ = *V*_del_ is at 0. If *V*_imm_ > / < *V*_del_, immediate choice probability is below/above 0.5. β regulates the steepness of the curve and thus the sensitivity toward differences in values. Lower β values require larger value differences (*x*-axis) to obtain a comparable probability (*y*-axis). **(D)** Discounted value (*y*-axis) for different delays (*x*-axis) for two hypothetical discount parameter values (κ = 0.05 in gray and κ = 0.005 in black). The colored dots represent the method's selected immediate rewards ( = *V*_imm_) at a given delay for the different induced immediate choice probabilities 0.3 (red), 0.5 (orange), and 0.7 (yellow). The distances between immediate values (colored dots) and delayed values (discounting curve) is constant across all delays to ensure equally induced probabilities across delays (see graph **(C)**). This also indicates that for subjects with different κ's, the reward and value ratios will vary. The left graph depicts selected rewards for a hypothetical β = 0.1 and the right graph for β = 0.4. While β regulates discounted value of the delayed reward *V*_del_, κ regulates the distance of the selected immediate reward around *V*_del_ with higher β resulting in smaller differences, making the differentiation between the two more difficult (that is, requiring higher sensitivity).

The designed temporal discounting paradigm went as follows: Participants were asked to solve an ICT in two separate runs. The ICTs consisted of both reward discounting and loss discounting trials presented within alternating blocks (see Figure 1A). The behavioral choices on the first run (referred to as “run A” in the following) were used to infer subject-level behavioral discounting models. These models were employed to generate the trials of the second run (referred to as “run B”; see also Figure 1B). Trials in run B were generated so as to elicit immediate choice probabilities (and correspondingly relative discounting frequencies) of 0.3, 0.5, and 0.7 in each participant. The probabilities were selected to obtain three behavioral gradings of low, medium, and high discounting probabilities. High and low discounting probabilities reflect “easy,” while 0.5 probabilities reflect “hard” trials in analogy to previous studies. In principle though, the probabilities are arbitrarily tunable.

This paradigm was assessed online and optimized in a series of experiments. After collecting data on one experiment (i.e., run A and run B), several alternative discounting models were separately inferred on the two runs and their ability to predict the behavior of the opposing run was assessed. The current model was then used to adjust and improve trials of run A, or a superior model was selected to update the trial-generating process for the successive experiment (see Figure 1B).

#### Run A

Individuals were instructed to choose between a smaller immediate or a larger later reward or loss. The magnitude of the delayed rewards and losses, as well as the delay duration was varied across trials. Each trial comprised a decision phase of up to 10 s (otherwise self-paced), as well as visual feedback of the selected choice and an inter-stimulus-interval of 1 s each.

In the initial experiment (exp 1), the delays were set to *D* = {2, 7, 30, 90, 180} days and the delayed outcomes to *r*_2_ = +/–{2, 5, 10, 20} £ (UK) for the reward and loss condition, respectively, following frequently used delays and delayed outcomes in previous studies (47, 71). Immediate outcomes were selected which - according to the hyperbolic discounting model - were expected to elicit an equal probability for immediate and delayed choices at different hypothetical discounting parameter values κ = {0.01, 0.1, 0.2, 0.6}, that is, to generate trials at the corresponding indifference points (see Equation 3 for details, where β was fixed to 1). Run A in exp 1 thus comprised 5 (delays) × 4 (delayed outcomes) × 4 (discounting parameters) × 2 (conditions: reward and loss) = 160 trials. Reward and loss trials were presented in blocks of 40 trials each. The trial order within blocks was fully randomized.

#### Run B

After completing run A, behavioral discounting models were inferred on the behavioral choices of each participant. We set out with the perhaps most commonly applied discounting model, the hyperbolic model, widely applied to study human choice in the ICT [e.g., (8, 9, 20, 36, 37, 44, 72–74)]. The model assumes that the values *V* for the delayed options *a*_2_ are discounted according to a hyperbolic function, that is, according to


(1) $$\begin{array}{r} V\left(a_{2}|s_{j}\right)\;=\;\left(\frac{1}{1\;+\;\kappa \;\cdot \;D}\right)r_{2}, \end{array}$$

while the values for the immediate options *a*_1_ correspond to the actual outcomes, *V*(*a*_1_|*s*_*j*_) = *r*_1_ (temporal delay *D* = 0 at this point). Here, the state *s*_*j*_ indexes the reward (*s*_1_) or loss (*s*_2_) condition, κ captures the inter-individual discounting degree (where high values indicate strong discounting), *D* the temporal delay, and *r*_*i*_ the actual outcome for the respective choice (*i* = 1 = immediate, *i* = 2 = delayed). We further refer to the factor in front of *r*_2_ which captures the devaluation strength as the discount factor.

While the majority of studies infer κ by fitting a sigmoid function to the behavioral performance under this model via least squares [see e.g., (31, 38, 43, 62, 63, 74)], we use the sigmoid to link the discount model to immediate choice probabilities and infer parameters via maximum likelihood estimation [see also (36, 75–80)]. The probability of an immediate choice *a*_1_ at any time *t* is given by


(2) $$\begin{array}{r} p\left(a_{1}|s_{j}\right)\;=\;\frac{1}{1{\;+\;e}^{\beta \left(V\left(a_{2}|s_{j}\right)-V\left(a_{1}|s_{j}\right)\right)}}, \end{array}$$

where β indicates the tendency to exploit (β → ∞) or explore (β → 0) choices (81), and *p*(*a*_2_|*s*_*j*_) = 1– *p*(*a*_1_|*s*_*j*_). This sigmoid is akin to a psychometric function used in psychology to map (differences in) stimulus intensity on to behavioral response probabilities, where here, we map differences in subjective values to the probability of an immediate response. Models were inferred (online) with constrained parameter optimization (using optimize.minimize() from the SciPy library, https://scipy.org/citing-scipy/, and κ ϵ [0, 10] and β ϵ [0, 100]).

The benefit of linking a sigmoid function to immediate choice probabilities is that we can rearrange Equation 2 and explicitly solve for immediate outcomes which elicit a predetermined choice probability in a given participant. Defining *p*_1_: = *p*(*a*_1_|*s*_*j*_) as the probability for the immediate choice (the one we want to adjust), and inserting the model values (Equation 1) into Equation 2, then rearranging for immediate outcomes *r*_1_, we obtain the condition


$$\begin{array}{rcl} r_{1}\; & = & \;V\left(a_{2}|s_{j}\right)\;+\;\frac{\log\left(\;\frac{p_{1}}{1\;-p_{1}}\;\right)}{\beta } \end{array}$$

(3) $$\begin{array}{rc} = & \;\left(\frac{1}{1\;+\;\kappa \;\cdot \;D}\right)r_{2}\;+\;\frac{\log\left(\;\frac{p_{1}}{1\;-p_{1}}\;\right)}{\beta } \end{array}$$

for the hyperbolic model (defined for 0 < *p*_1_ < 1). Intuitively, at *p*_1_ = 0.5, that is, if we want to induce an equal probability for an immediate and delayed option (to e.g., generate trials at the indifference points), the right part of Equation 3 drops such that the immediate value (and correspondingly the immediate reward) becomes equal to the discounted value. Increasing/decreasing immediate choice probability above/below 0.5, on the other hand, results in increasing/decreasing the immediate outcome. The condition in the middle further holds for all models which only differ in their expression of the discounted value. Note that we rearranged Equation 2 to solve for the immediate reward given an immediate choice probability *p*_1_ (see also Figures 1C,D for an illustration of the method's operating principle). One may however also apply this approach to solve for the appropriate delay (see Supplementary Methods S1 and Supplementary Figure S1).

Trials of run B were generated using this condition (Equation 3). Three trial types were defined, namely trials which were expected to evoke immediate choice probabilities of *p*_1_ = {0.3, 0.5, 0.7}, corresponding to trials in which we expected participants to mainly choose the delayed option (with *p*_1_ = 0.3), choose both options with equal probability (*p*_1_ = 0.5), or mainly choose the immediate option (with *p*_1_ = 0.7). Note that in the reward task *p*_1_ also corresponds to the discounting probability (as the immediate choice corresponds to the discounted choice) while for loss, the discounting probability is equal to *p*_2_ = 1 – *p*_1_ (as the delayed choice corresponds to the discounted choice).

For each choice probability, each delay *D*, and each delayed outcome *r*_2_ (as used in run A), immediate outcomes were thus determined according to the inferred subject specific model parameters κ and β. The initial run B thus comprised 5 (delays) × 4 (delayed outcomes) × 3 (choice probabilities) × 2 (condition: reward and loss) = 120 trials.

Note that a few parameter constellations could result in atypical trials with (1) negative immediate reward (corresponding to losses in reward trials) or positive immediate loss (corresponding to rewards in loss trials), (2) equal immediate and delayed reward/loss, or (3) larger immediate compared to delayed reward or smaller immediate compared to delayed loss. To avoid these trials, immediate outcomes were adjusted by iteratively increasing/ decreasing the delay durations by 1 until these cases were dissolved, or the minimum or maximum delay was reached. If still not dissolved, negative immediate rewards or positive immediate losses were set to 1 or -1 penny, while immediate rewards/losses which were equal to delayed rewards/losses were reduced/increased by 1 penny, respectively. All choice outcomes were hypothetical.

For the successive experiments, delays, outcomes, and discounting models were adapted to optimize the paradigm in agreement with the interim results (see section “RESULTS”).

### Sample

Healthy participants were recruited to participate in the online study via the Prolific website (https://www.prolific.co/). Eligibility criteria included age 18–65 and current residency in the United Kingdom (UK). Participants received £7.50 per hour as compensation for study participation. In total, 200 participants took part in the study (see Supplementary Table S1). Data were collected in batches of 50 individuals each. After each batch, the developed paradigm was evaluated and adjusted in line with the interim results and the proposed framework (see Figure 1B). Specifically, batch 1 and 2 were combined into one experiment (exp 1, *N* = 100), batch 3 represents the second experiment (exp 2, *N* = 50) and batch 4 represents the third experiment (exp 3, *N* = 50). Individuals were excluded from further analyses in case of not completing the first run, not completing the second run, or not responding to more than 10% of the trials during each run.

### Data Collection and Online Setup

The online study was programmed in JavaScript using the open-source package “jsPsych” (82) and was hosted on a custom virtual server using a Linux-Apache-PHP-MySQL stack (see Supplementary Figure S2). Model parameter inference and trial generation of run B was written in Python. All code needed for the setup and execution of the study can be found here: https://github.com/MathieuPinger/discounting-online.

Participants entered the study through a link on the Prolific website. Participant IDs were randomly generated for data storage. Additionally, a separate password-protected database associated each participant with a Prolific internal ID to ensure a study completion checkup.

After completing the consent form, participants filled out sociodemographic information (age, gender, education, employment, country of current residency). Subsequently, run A was presented, after which participants completed the alcohol use disorder identification questionnaire (AUDIT; (83)) and the short version of the Barratt-Impulsiveness-Scale (BIS-15; (84)). During this time, subject-level behavioral models were inferred on data from run A, and used to generate trials for run B which was presented immediately after the questionnaires.

The study was approved by the ethics committee of the Medical Faculty Mannheim, University of Heidelberg (2019-633N).

### Data Analysis

#### Behavioral Models and Model Parameters

The initial experiment was conducted with the most frequently used delay discounting model in human research, the hyperbolic discounting model (see Equation 1). The model was compared with several other proposed models in the field. These models differ in the assumption of how an individual devaluates the delayed outcome (see Equation 1). For completeness, the compared models include

- The hyperbolic model (20, 85), where $V\left(a_{2}|s_{j}\right)\;=\left(\frac{1}{1\;+\;\kappa \;\cdot \;D}\right)r_{2},$ with κ ϵ [0, ∞).
- The exponential model (68), where $V\left(a_{2}|s_{j}\right)\;=\;\kappa^{D}r_{2}$, with κ ϵ [0, 1], implying that the perceived value of a delayed outcome is discounted exponentially scaled by the individual discounting rate κ.
- The quasi-hyperbolic model [also known as the beta-delta model; (5, 69)], where $V\left(a_{2}|s_{j}\right)=\gamma \;\kappa^{D}r_{2}$, for *D* > 0, with γ, κ ϵ [0, 1], where the exponential discounting of the delayed outcome is additionally modulated by a second linear discount parameter γ.
- The hyperboloid model (27, 65), where $V\left(a_{2}|s_{j}\right)\;=\;\frac{1}{{\left(1\;+\;\kappa \;\cdot \;D\right)}^{s}}\;r_{2},$ with κ ϵ [0, ∞) and *s* ϵ [0, 1], similar to the hyperbolic discounting model, only that the discounting factor is scaled by an additional parameter *s*.
- The modified hyperboloid model (20, 86), where $V\left(a_{2}|s_{j}\right)\;=\;\frac{1}{\left(1\;+\;\kappa \;\cdot \;D^{s}\right)}\;r_{2}$, with κ ϵ [0, ∞) and *s* ϵ [0, 1], which is a slight modification of the hyperboloid model, suggesting that *s* solely scales the delay and thus may account for differences in perceived time.
- The double-exponential model (87), where $V\left(a_{2}|s_{j}\right)\;=\;\left({w\kappa_{1}}^{D}\;+\;\left(1-w\right){\kappa_{2}}^{D}\right)r_{2}$, with *w*, κ_*i*_ϵ [0, 1], which is inspired by the evidence that choices result from the competition between two neurobiological systems (referred to as valuation and control) scaled by their own decay rates (κ_1_ and κ_2_), each contributing by a factor *w* and 1 – *w*, respectively, and,
- The constant-sensitivity model (64), where $V\left(a_{2}|s_{j}\right)\;=\;exp\left(-{\left(\;\kappa \cdot D\right)}^{\delta }\right)\;r_{2}$, with κ, δ > 0. This model accounts for decision heuristics by including the κ parameter as an indicator of impatience, and δ reflecting time sensitivity. Note that this model differs from model (3) in terms of parameter constraints.

Models were compared by inferring each model on each experimental run (A and B) and condition (reward and loss) of each participant and using the inferred parameters to assess the out-of-sample prediction error (PE) on the respective contrary run (i.e., predicting behavior in B when inferring models on A and vice versa). The PE here was defined as 1–${\hat{p}}_{j}$, where ${\hat{p}}_{j}\;=\;\frac{1}{T}\overset{T}{\underset{t}{\sum }}p\left(a_{t}|s_{j}\right)$, i.e., is defined as the average over the predicted probabilities of observed choices per condition *j*. For simpler interpretability, only ${\hat{p}}_{j}\;$is reported.

Note that the predicted probability will depend on trial difficulty where more difficult choices (i.e., trials closer to the indifference point of a subject), should by definition be predicted with a lower probability. We thus do not expect an average predicted probability close to 1 in either run. Particularly in run B, where by condition we generate trials eliciting immediate choice probabilities of 0.3, 0.5, and 0.7, the expected prediction should lie around (0.7 + 0.5 + 0.7)/3 = 0.63 (see also Supplementary Figure S5 right), and may slightly deviate due to slight trail adjustments (see Section Run B) or to using a model not used for trial generation.

#### Behavioral Variables and Data Analysis

Temporal discounting was measured by assessing the frequency of discounted choices for each run, each condition, and each manipulated (immediate choice) probability (cf. *p*_1_), as well as the median reaction time (RT) for these conditions. Individuals which discounted in < 5% of all trials were defined as “non-discounters.”

We further assessed subjective impulsivity by averaging across all items of the BIS-15 (i.e., BIS-total), as well as across items related to the three sub-scales, namely attentional impulsivity (i.e., the difficulties to focus attention or concentrate), motor impulsivity (i.e., acting without thinking), and non-planning impulsivity (i.e., lack of future orientation), respectively (84). We also assessed abusive or harmful alcohol consumption by the alcohol use disorders identification test (AUDIT; (83)).

Model parameters and behavioral variables such as discounting parameters, choice frequencies, as well as (absolute) deviations between observed and expected choice frequencies, were compared via *t*-tests for paired or unpaired samples (i.e., for comparisons between conditions and runs vs. comparisons between experiments; please note, absolute deviations were used when comparing experiments) in case of normally distributed variables, or nonparametric Wilcoxon signed-rank tests for paired and Wilcoxon rank-sum tests for unpaired samples in case of normality violation. Variables were correlated via Pearson's or Spearman's correlation coefficient, respectively. The number of discounters vs. non-discounters across experiments was compared via Chi-square tests for equal and Fisher's exact test for unequal sample sizes. Statistical significance was set to *p* < 0.05 (two-tailed) for all tests. Individuals repeating either option in more than 95% of all trials during run A, making it difficult to obtain valid parameter estimates, were removed from analyses on run B where deemed necessary (explicitly mentioned in the Results Section). Individuals with extreme discounting parameters κ > 2 were removed from all analyses related to this parameter.

## Results

### Experiment 1

Two separate batches of 50 individuals each were collected for exp 1. After collecting the first sample (*N* = 50), we observed a minor bug in the paradigm code which resulted in the generation of run B trials with equal immediate and delayed outcomes. These trials occurred in < 1.2% of all trials (around 2–3 trials in 24 participants). We thus immediately collected a second sample (*N* = 50) with this bug fixed and removed the afore-mentioned trials from the first sample in the behavioral measures analyses. Two individuals were excluded from further analyses since they had > 30% missing values in one condition. Exp 1 thus included *N* = 98 individuals.

**Figure 2:**
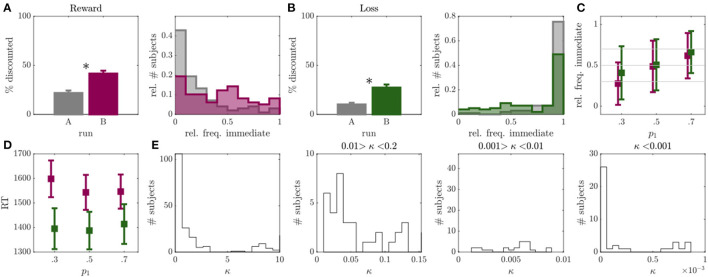
Results of experiment 1. **(A)** Left: Percentage of discounted choices in run A (gray) and run B (magenta) for reward condition. Right: Histograms over relative frequency of immediate choices in run A (gray) and run B (magenta) for reward condition. Asterisks indicate significant differences. **(B)** Same as A for loss condition with run B displayed in green. **(C)** Mean and standard deviation of observed relative frequency of immediate choices for the experimentally evoked probabilities *p*_1_ = {0.3, 0.5, 0.7} (*x*-axis) in run B (individuals with >95% or <5% immediate choices were removed, *N* = 28 or 29% in reward, *N* = 52 or 53% in loss condition). **(D)** Mean and standard deviation of median reaction time (RT; *y*-axis) for *p*_1_ = {0.3, 0.5, 0.7.} trials (*x*-axis) in run B for reward (magenta) and loss (green) conditions. **(E)** Histograms over discounting parameter κ of the hyperboloid model for both loss and reward conditions, displayed at different resolutions and bin widths.

As reported in multiple other studies [e.g., 31, 33–35, 38–40], we observed a high percentage of individuals, namely 58%, which showed no temporal discounting in at least one condition of the initial run A (see Figures 2A,B). This was particularly evident for the loss discounting condition which yielded 53% of non-discounters (see Figure 2B; non-discounters being defined as individuals which discounted in < 5% of all trials, cf. Behavioral Variables and Data Analysis).

After adapting the experimental trials to the individual participants in run B, we observed a considerable reduction in non-discounters (from 26 to 13% in the reward, and from 53 to 42% in the loss condition), and a significant increase in the frequency of discounted choices in both reward (*Z* = 5.06, *p* < 0.001; see Figure 2A), and loss (*Z* = 4.85, *p* < 0.001; see Figure 2B) conditions. This was accompanied by a significant increase in the inferred discount parameters κ, signaling higher discounting (reward: *Z* = 2.83, *p* = 0.005, loss: *Z* = 4.57, *p* < 0.001).

The observed choice frequencies in run B, moreover, aligned with the experimentally manipulated probabilities. That is, the frequency of immediate choices increased in response to trials with *p*_1_ = 0.7 compared to *p*_1_ = 0.5 (reward: *Z* = 5.43, *p* < 0.001; loss: *Z* = 4.91, *p* < 0.001), and to trials with *p*_1_ = 0.5 to *p*_1_ = 0.3 (reward: *Z* = 6.21, *p* < 0.001; loss: *Z* = 3.72, *p* < 0.001). However, the observed choice frequencies deviated significantly from the model expectations w.r.t. all three trial types in the loss condition (*p*_1_ = 0.3: *Z* = 6.52, *p* < 0.001; *p*_1_ = 0.5: *Z* = 5.63, *p* < 0.001; *p*_1_ = 0.7: *Z* = 4.27, *p* < 0.001), as well as for *p*_1_ = 0.7 in the reward condition (*Z* = 3.35, *p* < 0.001; other comparisons *p* > 0.05). This was somewhat due to individuals who consistently chose only one option showing no behavioral variation in general (concerning*N* = 28 in the reward and*N* = 52 in the loss condition). After removing these individuals from the analysis, the mean of the choice frequency distributions moved closer to the model expectations (see Figure 2C), although still significantly deviating for *p*_1_ = 0.7 in the reward (*Z* = 3.35, *p* < 0.001) and for *p*_1_ = 0.3 in the loss condition (*Z* = 2.21, *p* = 0.027; all other *p'*s>0.05). We did not observe an increase in RT toward *p*_1_ = 0.5 trials (defined as “hard” trials in the field) as compared to the other two trial types (amounting to “easy” trials here; reward: *p*'s > 0.329; loss: *p*'s > 0.290; see also Figure 2D).

In conclusion, the first experiment indicated that by applying the condition in Equation 3, we were able to reduce the number of non-discounters and evoke higher discounting frequencies. We could also show that for individuals which generally showed behavioral variation in run A, the observed immediate choice frequencies on average largely centered around the model expectations in run B. However, the standard deviation of these choice frequencies was rather high. Also, RT's did not reflect a clear separation between ‘hard' and ‘easy' trials (see Figure 2D).

Two possible (non-exclusive) explanations may account for these findings. First, the hyperbolic model may not have captured the entire systematic data variation, such that the model predictions and thus the generated model-based (run B) trials were somewhat biased. In fact, the hyperbolic model performed worse in predicting (out-of-sample) behavior than several other tested models (see Supplementary Figure S3), achieving a prediction of only 0.55 for both reward and loss (as compared to predictions > 0.7, see Supplementary Figure S3). Second, going one step back, trials in run A may not have evoked enough behavioral variability to infer valid model parameters required to generate subject specific trials. Since the percentage of discounted choices during run A was rather low for both reward and loss conditions (Figures 2A,B), and we obtained a higher behavioral model agreement after excluding individuals with low behavioral variability from analysis (Figure 2C), the second explanation seemed rather likely. A poor (hyperbolic) model fit could therefore also be due to a poor selection of run A trials. As an initial step to further improve the paradigm, we thus first focused on improving trials of run A to promote valid parameter inference, before altering the underlying trial-generating model.

#### Modification

Trials of run A were initially generated by using common delays and delayed outcomes found in the literature and finding the indifference points to these values, given a set of hypothetical discounting parameters κ (cf. Section Run A). To improve this run, we now focused on generating trials which more closely matched the actually observed discounting parameters and behavior in run B (since we observed more discounting in this run). We observed a bimodal κ distribution, with the majority of individuals being characterized by κ's ranging between 2.6 × 10^−11^ and 3, and a few above 7 (see Figure 2E left). The dominance of the left mode indicates that most participants were characterized by rather low discounting rates (see also Figures 2A,B), and, in particular, far lower than the ones used for the initial run generation (cf. Run A). Run A was thus modified to better represent the left mode of the actually observed κ distribution (see Figure 2E for a high resolution of the true κ distribution). Around half the sample was characterized by a κ < 0.2 (reward: *N* = 67; loss: *N* = 62). Of these, 20 participants in the reward and 16 participants in the loss condition exhibited κ'*s* between 0.01 and 0.2, 17 participants in the reward and 16 participants in the loss condition ranged between 0.001 and 0.01, and 20 participants in the reward and 21 participants in the loss condition were characterized by κ'*s* < 0.001 (among which 10 in the reward and 16 participants in the loss condition were characterized by κ'*s* < 0.00001; see Figure 2E). To cover this range of the parameter distribution, we updated the set of hypothetical discounting parameters in run A (cf. Section Run A) to κ = {0.00001, 0.001, 0.01, 0.6}.

We further exchanged the shortest delay (2 days delay) with a long delay (365 days delay) as longer delays additionally encourage discounting (cf. Equation 3) such that the new set of delays was set to *D* = {7, 30, 90, 180, 365}. Lastly, we removed the lowest delayed outcome and replaced it by a higher delayed outcome such that the new set of delayed rewards and losses was *r*_2_ = +/–{5, 10, 20, 50}£.

### Experiment 2

**Figure 3:**
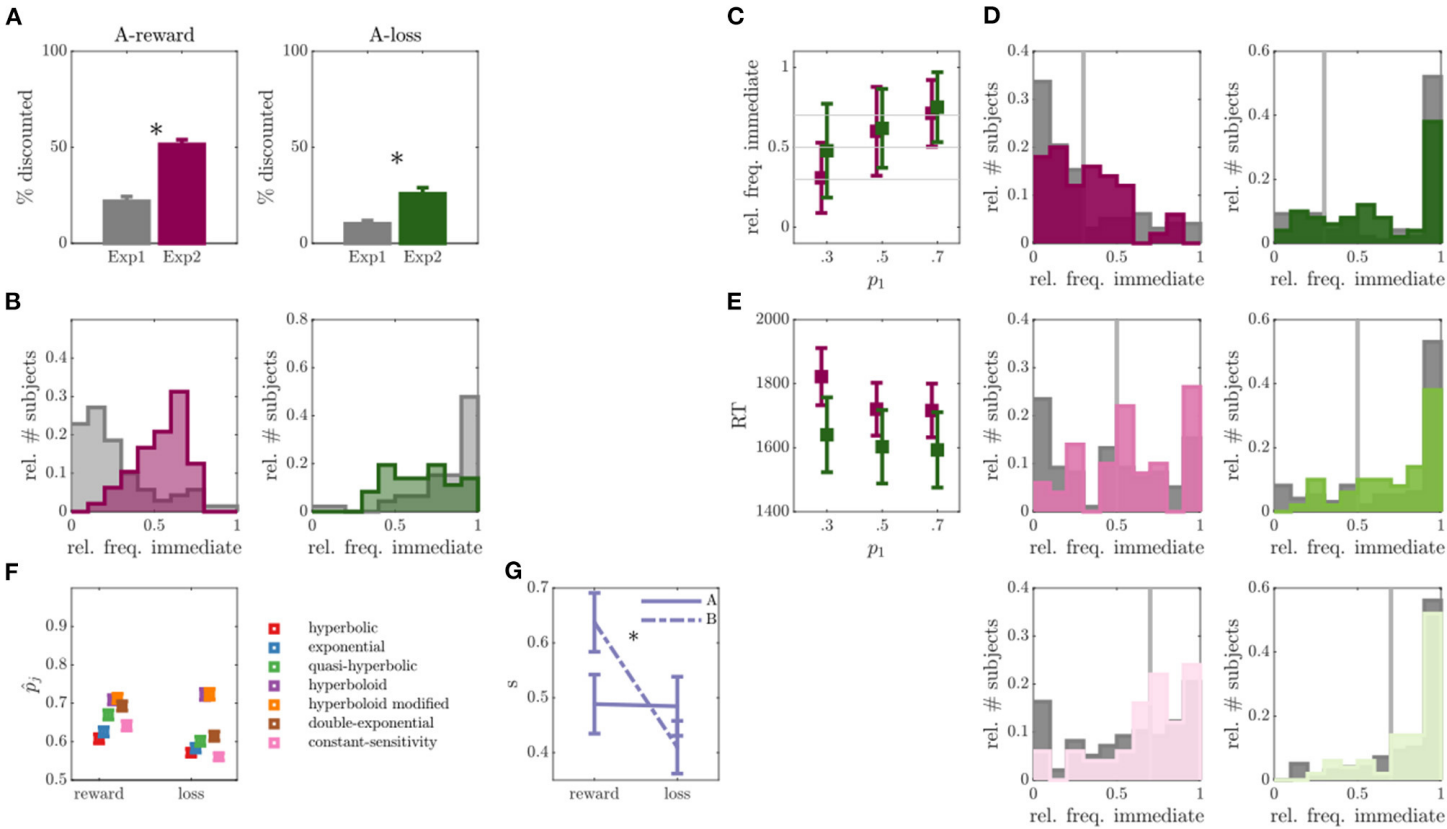
Results of experiment 2. **(A)** Percentage of discounted choices in run A for experiment (exp) 1 and exp 2 for reward (left) and loss (right) discounting. Asterisks indicate significant differences. **(B)** Empirical distribution of the relative frequency of immediate choices for reward (left) and loss (right) conditions in run A of exp 1 (gray) and exp 2 (colors). **(C)** Relative frequency of immediate choices (*y*-axis) in run B of exp 2 as a function of experimentally manipulated probabilities (*x*-axis) for reward (magenta) and loss (green) conditions (individuals without behavioral variability were removed, *N* = 2 for reward, *N* = 14 for loss condition). **(D)** Empirical distributions of the relative frequency of immediate choices in run B for trials with immediate choice probability 0.3 (top), 0.5 (middle) and 0.7 (bottom), as also indicated by the gray line. Reward condition is displayed left, loss right, exp 1 in gray and exp 2 in color. **(E)** Average over median reaction times (RT) for the three experimentally manipulated immediate choice probabilities for reward (magenta) and loss (green) conditions. **(F)** Average predicted (out-of-sample) probability of observed responses ${\hat{p}}_{j}$ (*y*-axis) for reward and loss conditions (x-axis) for different models. **(G)** Inferred scaling parameters *s* of the modified hyperboloid model for reward and loss discounting conditions.

Fifty individuals completed exp 2 with altered trials of run A. In run A of exp 2, compared to exp 1, we observed a considerably lower percentage of non-discounters in the reward condition (*N* = 2, that is, a drop from 26% to 4%; OR = 8.67, *p* < 0.001), as well as in the loss condition (*N* = 14, a drop from 53% to 28%; OR = 2.91, *p* = 0.005) (see also Figure 3A). The average percentage of discounted choices also significantly increased in run A of exp 2 compared with run A of exp 1, for both reward and loss conditions (reward: *Z* = 6.58, *p* < 0.001; loss: *Z* = 3.93, *p* < 0.001; see Figures 3A,B). In fact, for the reward condition it amounted to 51%, rendering more optimal conditions for parameter inference. In the loss task, this percentage remained lower, however, with around 26%. In both conditions, we furthermore observed a significant increase in RT compared with exp 1 (reward *Z* = 4.47, *p* < 0.001; loss: *Z* = 4.26, *p* < 0.001), suggesting that choices became more difficult, closer to the indifference points of each participant. We conclude that by model based adaptation of run A, we were able to reduce the number of non-discounters and increase behavioral variability within participants (see also Figures 3A,B).

Regarding run B, the observed immediate choice frequencies in the reward condition centered around the model expectations (see Figure 3C; reward: *p*_1_ = 0.3: *Z* = 0.50, *p* = 0.615; *p*_1_ = 0.5: *Z* = 1.46, *p* = 0.145; *p*_1_ = 0.7: *Z* = 0.09, *p* = 0.923), while still significantly deviating in the loss condition (*p*_1_ = 0.3: *Z* = 4.93, *p* < 0.001; *p*_1_ = 0.5: *Z* = 4.55, *p* < 0.001; *p*_1_ = 0.7: *Z* = 3.22, *p* = 0.001). Nonetheless, for both reward and loss conditions, the absolute deviations between expected and observed relative immediate choice frequencies were lower in exp 2 compared with exp 1 (statistically significant for reward: *p*_1_ = 0.3: *Z* = 2.64, *p* = 0.008; *p*_1_ = *0.5: Z* = *1.67, p* = *0.096*; *p*_1_ = 0.7: *Z* = 2.65, *p* = 0.008; and loss: *p*_1_ = 0.5: *Z* = 2.24, *p* = 0.025; see Figure 3D), suggesting an improvement in the proposed paradigm. However, many non-discounters remained in the loss condition of run B (*N* = 14, Figure 3D).

Given that run A now rendered better conditions for parameter inference, we next focused on evaluating and improving the paradigm underlying model. For this, we inferred several discounting models suggested by the literature on run A and run B separately (cf. Section Behavioral Models and Model Parameters) and assessed their ability to predict the behavior in the opposing run, that is, inferring parameters on run A and predicting behavior in run B and vice versa. The two experimental runs thus allowed us to assess an estimate of the out-of-sample PE which is less biased and preferred over in-sample estimates (70, 88), commonly used in the field [e.g., (38, 61, 89)]. Figure 3F shows the model comparison results averaged over predictions in both runs. The hyperboloid and the modified hyperboloid model outperformed all other models in both reward and loss conditions, with a slight preference for the modified hyperboloid model (20, 86). On average, the modified hyperboloid model predicted responses successfully with 0.71 probability in the reward, and 0.72 probability in the loss condition. In contrast, the most commonly used hyperbolic and exponential models performed comparatively poorly (exponential model: ${\hat{p}}_{reward}$ = 0.64, loss ${\hat{p}}_{loss}$ = 0.58, hyperbolic model: ${\hat{p}}_{reward}$ = 0.61, ${\hat{p}}_{loss}$ = 0.57; see also Figure 3F and Supplementary Figure S6). These results held true when evaluating a weighted PE where the response probability was averaged over predictions for immediate and delayed choices (ensuring that predictions were not only good in predicting a dominant response, sometimes referred to as the majority class, see Supplementary Figure S4). Note that the hyperboloid models also outperformed the exponential and hyperbolic models on predicting the data of exp 1 (see Supplementary Figure S3).

Evaluating the parameters of the modified hyperboloid model (cf. Behavioral Models and Model Parameters) also revealed some interesting insights into behavior. The additional scaling parameter *s* distinguishing this model was extremely reliable, observed in terms of a high correlation in *s* between runs (for reward: *r* = 0.44, *p* = 0.001; for loss: *r* = 0.44, *p* = 0.001) and conditions (run A: *r* = 0.26, *p*=0.064; run B: *r* = 0.24, *p*=0.091), and pointing toward a trait like scaling of delay. Apart from that, *s* was higher in the reward compared with the loss condition (*Z* = 3.43, *p* < 0.001; see Figure 3G).

#### Modification

Following these results, we updated the paradigm to now generate trials of run B according to the modified hyperboloid model (20, 86). The lower values in the scaling parameter *s* observed for the loss condition effectively reduce discounting (by shrinking the delay duration). To further encourage discounting in the loss task, we therefore also exchanged the shortest delay (7 days delay) with a long delay (3 years) in the loss condition only. The new set of delays for the loss condition was set to *D* = {30, 90, 180, 365, 1095}.

### Experiment 3

Fifty individuals completed exp 3 with altered trials of run A in the loss condition and an altered trial-generating discounting model for run B (now using the modified hyperboloid model).

**Figure 4:**
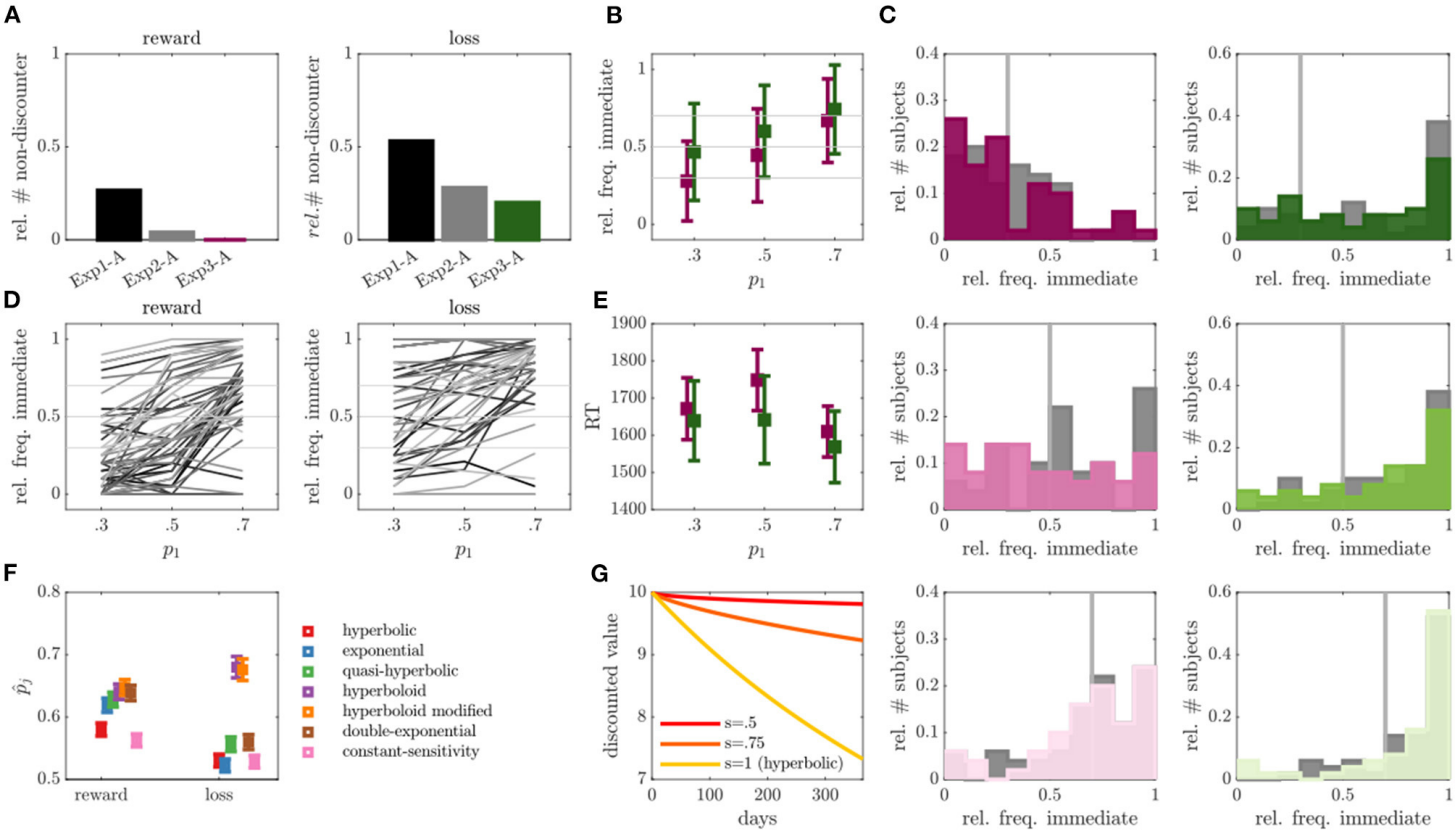
Results of experiment 3. **(A)** Relative number of non-discounter in run A across experiments for reward (left) and loss (right) conditions. **(B)** Relative frequency of immediate choices (*y*-axis) in run B of exp 3 as a function of experimentally manipulated probabilities (*x*-axis) for reward (magenta) and loss (green) conditions (individuals without behavioral variability were removed, *N* = 0 for reward, *N* = 10 for loss condition). **(C)** Empirical distributions of the relative frequency of immediate choices in run B for trials with immediate choice probability 0.3 (top), 0.5 (middle) and 0.7 (bottom), as also indicated by the gray line. Reward condition is displayed left, loss right, exp 2 in gray and exp 3 in color. **(D)** Average relative frequencies of immediate choices per subject across the three experimentally manipulated immediate choice probabilities. **(E)** Average over median reaction times (RT) for the three experimentally manipulated immediate choice probabilities for reward (magenta) and loss (green) conditions. **(F)** Average predicted (out-of-sample) probability of observed responses ${\hat{p}}_{j}$ (*y*-axis) for reward and loss conditions (*x*-axis) for different models averaged over run A and B. **(G)** Hypothetical discounting curves in the modified hyperboloid model for κ = 0.01, *r*_1_ = 10, and different values of scaling parameter *s*.

In run A, we observed a slight reduction in the number of non-discounters compared with exp 2, with 0 non-discounters observed in the reward and 10 non-discounters observed in the loss condition, although this was statistically not significant (*p* > 0.875; see Figure 4A). The average frequency of discounted choices did also not significantly differ in run A of exp 2 compared with run A of exp 3, neither for the reward (*Z* = 1.08, *p* = 0.277), nor for the loss condition (*Z* = 0.33, *p* = 0.740). We observed an average of 48% discounted choices in the reward and 27% in the loss condition in exp 3.

In run B, we also observed a slight, but statistically not significant reduction in the number of non-discounters (reward: *N* = 2 or 4%; loss: *N* = 12 or 24%). The observed immediate choice frequencies again increased with increasing model expectations (i.e., from 0.3 to 0.5, and from 0.5 to 0.7) both on average (all *p*'s < 0.001), as well as (largely) on a single subject level (see Figures 4B,D). For the reward condition, the observed frequencies seemed to moreover center around the model-based expectations (see Figure 4B; *p*_1_ = 0.3: *Z* = 0.88, *p* = 0.378; *p*_1_ = 0.5: *Z* = 1.23, *p* = 0.221; *p*_1_ = 0.7: *Z* = 0.37, *p* = 0.712), while still deviating significantly for the loss condition (see Figure 4B; *p*_1_ = 0.3: *Z* = 4.27, *p* < 0.001; *p*_1_ = 0.5: *Z* = 3.60, *p* < 0.001; *p*_1_ = 0.7: *Z* = 3.31, *p* < 0.001). The absolute deviations between observed and expected immediate choice frequencies were again lower than those in exp 1, indicating choice frequencies were more consistent with model expectations (statistically verifiable for reward *p*_1_ = 0.7: *Z* = 2.67, *p* = 0.008; loss *p*_1_ = 0.3: *Z* = 2.05, *p* = 0.040; *p*_1_ = 0.5: *Z* = 1.89, *p*=0.059), but remained comparable to those in exp 2 (that is, no significant differences were observed, *p*'s > 0.2; see also Figure 4C). In contrast to exp 1 and 2, RTs were however more in line with theoretical expectations by which RT increases toward “harder” trials (see Figure 4E and Figure 3E in comparison). Individuals responded slower to reward trials close to the indifference point (i.e., *p*_1_ = 0.5) as compared to trials far from the indifference point (*p*_1_ = 0.7: *Z* = 3.20, *p* = 0.001; *p*_1_ = 0.3: *Z* = 1.70, *p* = 0.089). Although this was statistically not verifiable for the loss condition (*p*'s > 0.237), a qualitatively consistent picture was observed (see Figure 4E).

The out-of-sample based model comparison analysis suggested once more that the hyperboloid models outperformed all other tested models in both the reward and loss conditions (see Figure 4F; hyperboloid model ${\hat{p}}_{reward}$ = 0.64 and ${\hat{p}}_{loss}$ = 0.68, modified hyperboloid model ${\hat{p}}_{reward}$ = 0.65 and ${\hat{p}}_{loss}$ = 0.68). Similar to exp 2, the hyperbolic and exponential models performed rather poorly, particularly in the loss condition (hyperbolic model ${\hat{p}}_{reward}$ = 0.58 and ${\hat{p}}_{loss}$t = 0.53, exponential model ${\hat{p}}_{reward}$ = 0.62 and ${\hat{p}}_{loss}$ = 0.52). Once more, the scaling parameter *s* was lower in the loss compared with reward condition in run B (*Z* = 3.17, *p* = 0.002).

### Joint Analysis of Experiments 1, 2, and 3

**Figure 5:**
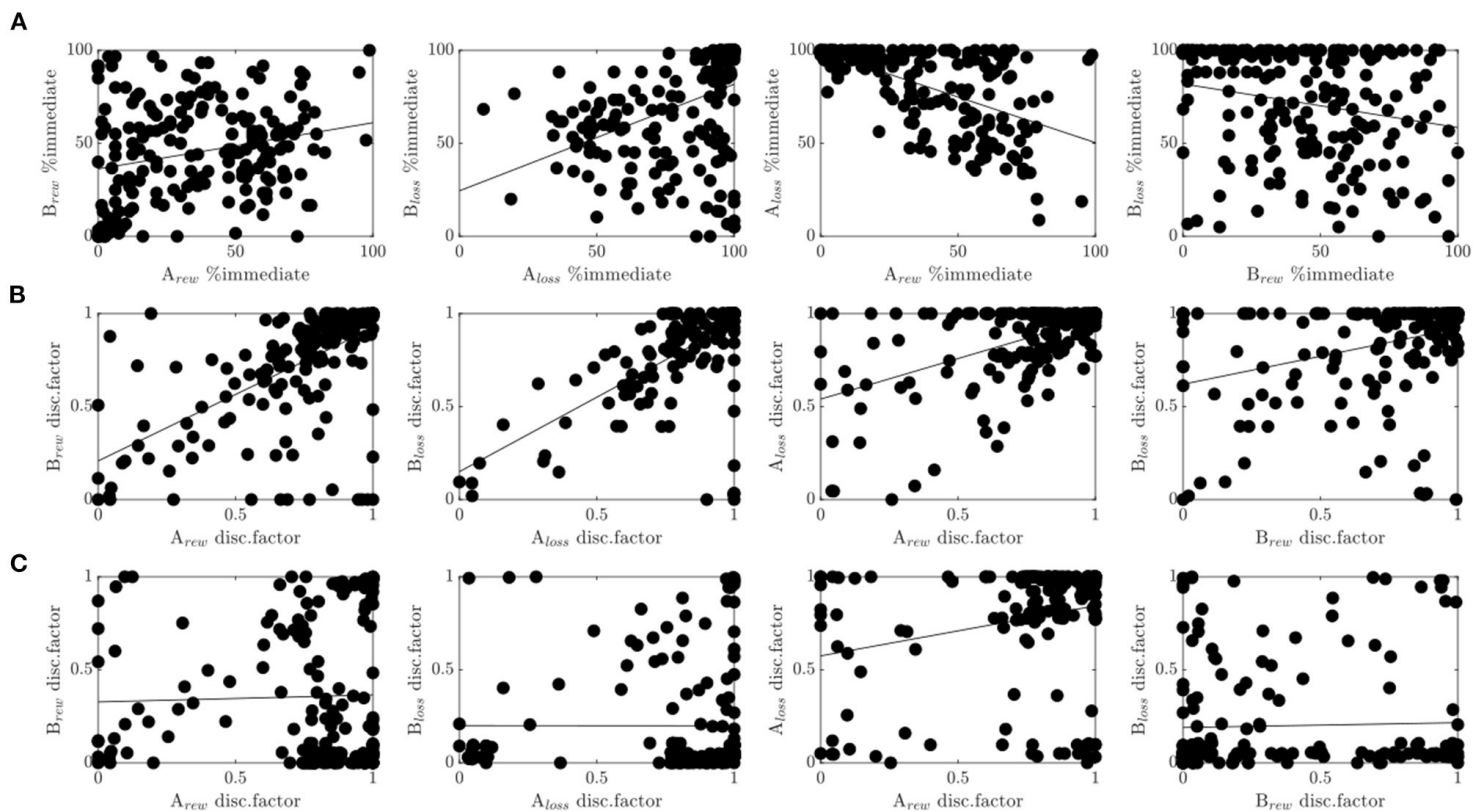
Cross-experimental results. **(A)** Correlations of the percentage of immediate choices between run A and run B for the reward condition (1st plot), and the loss condition (2nd plot), and between reward and loss conditions within run A (3rd plot), and run B (4th plot). **(B)** Same as in **(A)** only with the discount factor evaluated at delay *D* = 30 for the hyperboloid model. **(C)** Same as in **(B)** for the hyperbolic model.

Lastly, we investigated correlations between behavioral variables and model parameters across all three experiments to gain a deeper understanding of involved mechanisms during reward and loss discounting. First of all, there was a moderate correlation between the immediate choice frequencies of run A and B (reward: *r* = 0.24, *p* = 0.001, loss: *r* = 0.42, *p* < 0.001, see Figure 5A) suggesting at least some reliability in delay discounting processes as assessed in terms of choice frequency. Second, there was a considerable (expected negative) correlation between loss and reward (run A: *r* = −0.59, *p* < 0.001, run B: *r* = −0.22, *p* = 0.002, see Figure 5A), suggesting commonalities in the processing of reward and loss discounting.

In agreement with these results, the discount factor of the modified hyperboloid model (evaluated at delay *D* = 30) was highly correlated across runs and conditions (see Figure 5B). We observed a considerable correlation between run A and run B (reward: *r* = 0.6, *p*<0.001; loss: *r* = 0.65, *p*<0.001), and between reward and loss conditions (run A: *r* = 0.53, *p* <0.001, run B: *r* = 0.35, *p*<0.001). The correlations assessed on the discount factors were even higher than when assessed on the choice frequencies (*Z*'s>3.24, *p*'s<0.001). Discounting parameters κ were similarly correlated across runs (reward: *r* = 0.43, *p* < 0.001; loss: *r* = 0.48, *p* < 0.001) and conditions (run A: *r* = 0.37, *p* < 0.001; run B: *r* = 0.46, *p* < 0.001), although significantly less so (*Z*'s > 1.97, *p*'s < 0.024), with exception of the correlation between conditions during run B (*Z* = 1.21, *p* = 0.112).

In contrast, the discount factor of the hyperbolic model (evaluated at delay *D* = 30) did not correlate across runs (reward: *r* = 0.03, *p* = 0.698; loss: *r* = 0, *p* = 0.991, see Figure 5C). It correlated moderately between reward and loss conditions of run B (*r* = 0.24, *p* < 0.001), but not run A (*r* = 0.03, *p* = 0.633). The correlations observed for the hyperbolic model were therefore also significantly lower than the ones observed for the hyperboloid model (*Z*'s > 3.33, *p's* < 0.001). A qualitatively similar picture held true when evaluating the discount parameter κ which is proportional to the discount factor in the hyperbolic model. These results suggest that cognitive processes related to delay discounting were only captured reliably in the superior model, that is, the model with superior prediction performance. Note that the scaling parameter *s* of the hyperboloid model was also reliable, that is, correlated across reward and loss conditions (run A: *r* = 0.19, *p*=0.006, run B: *r* = 0.25, *p*<0.001), and across runs (reward: *r* = 0.31, *p* < 0.001; loss: *r* = 0.26, *p* < 0.001).

We also observed several differences between reward and loss conditions. The discount parameters κ and the scaling parameters *s*, were higher in the reward compared with the loss condition (κ run A: *Z* = 5.72, *p* < 0.001; κ run B: *Z* = 2.71, *p* < 0.001; *s* run A: *Z* = 1.99, *p* = 0.046; *s* run B: *Z* = 5.53, *p* < 0.001), while the discount factor was lower in the reward condition (run A: *Z* = 7.05, *p* < 0.001; run B: *Z* = 5.53, *p* < 0.001). Note though that despite the parameter constraints on scaling parameter *s*, we did observe moderate correlations between *s* and κ in the reward condition (run A: *r* = −0.31, *p* < 0.001; run B: *r* = −0.31, *p* < 0.001), suggesting slight issues with parameter identifiability.

W.*r*.t. subjective reports, we did not observe any associations between model parameters and subjective reported impulsivity or alcohol use behavior (*p*'s > 0.147). We did also not observe any correlations between subjective reports and the discount factors of the hyperbolic model (all *p*'s > 0.105). Exploratory analyses revealed a weak negative association between the loss discounting factor of the modified hyperboloid model (evaluated at *D* = 30) in run A and impulsivity (BIS-total: *r* = −0.15, *p* = 0.037), and between the loss discounting factor of the modified hyperboloid model in run B and alcohol use behavior (AUDIT-total: *r* = −0.14, *p* = 0.044; see Supplementary Figure S7).

## Discussion

A long-standing problem with the experimental measurement of cognitive functions based on group statistics is that an identical experimental trial presented to different subjects may elicit very different levels of functioning due to high inter-individual variability [(90–94); see also (95)]. This aggravates the reliable measurement of cognitive mechanisms and limits the comparability of results between subjects. For example, the same aversive stimulus in a fear conditioning paradigm can lead to very different degrees of fear association across individuals (93, 94). To remedy this problem, a common approach is to adapt experimental conditions such as stimulus intensities to the subject, making the experimental condition more comparable and less heterogenous across individuals (94, 96). Similarly, in delay discounting, the extent of discounting behavior is known to vary widely between subjects [(97, 98); for review see (25, 26)]. In an ICT, adjusted experimental settings for delays and outcome values per subject are therefore required to map a similar magnitude of discounting between subjects (31, 32, 36, 43, 53, 54), whereby poorly adaptive or non-adaptive experimental designs may even lead to subjects entirely not discounting. This may result in the exclusion of these subjects from further analyses [similar to conditioning paradigms (94)]. Here, we attempt to address this problem and propose a general approach to tailor experimental trials to the single subject. The underlying idea of this approach is that by modeling behavior as being probabilistically generated by the experiment and the cognitive function of interest, we can use the model to alter experimental components so as to align behavior. Besides reducing variance between subjects within an experimental condition, the proposed approach offers an additional advantage to current adaptive designs. It allows to generate trials associated with the entire range of discounting probabilities thus enabling to measure graded levels of discounting behavior. Both the model and the experimental components are optimized in an iterative process. We apply the proposed approach here to reward and loss delay discounting.

Our experimental paradigm is divided into two runs, run A and run B, which both consist of an identically structured delay discounting task which differs only in the prompted outcomes and delays (but could also be extended to other tasks and processes). From the behavioral results of run A, we infer subject-level models that probabilistically explain each participant's behavior. The modeled behavioral probabilities are then used to design (that is, solve for) experimental trials of run B to elicit discounting behavior with a predetermined probability. Here, we chose trials that, according to the applied model, should elicit a probability for the discounted option of 0.3, 0.5, and 0.7, although the approach principally allows for an arbitrary grading. The behavior in run B was then in turn used to (i) optimize run A based on the current model and (ii) evaluate and adjust the model-by-model comparison analyses. We tested the protocol in three sequential experiments.

Overall, we were able to significantly reduce the number of individuals showing no behavioral discounting. In addition, we were able to largely induce graded levels of discounting behavior on a single subject level. That is, the observed frequency of immediate choices in both the reward and the loss condition increased within participants with increasing immediate choice probability predicted by the behavioral model. In the reward task, this choice frequency was not only graded, but on average also consistent with the specific model expectation.

The match between model expectation and behavior improved across the successive experiments. In the first experiment we observed that the participants' behavior in run B was graded with respect to the predetermined probabilities, although the actual deviation from these probabilities was rather high. We also observed a high number of non-discounters in both conditions. By model-based adjustment of run A trials, we were able to drastically reduce this number in experiment 2, an issue commonly reported in the delay discounting literature, whereby studies report various rates of non-systematic discounting behavior ranging from 7% up to 50% of the investigated samples (31, 35–45, 89). In addition, the adjustments led to higher behavioral variability within participants, rendering better conditions to validly infer model parameters in run A. This in turn resulted in lower deviations between observed behavior and model predictions in run B of exp 2. Our procedure therefore successfully generated graded response conditions with lower variance, that is, higher behavioral homogeneity within conditions of exp 2.

After systematic model comparison analyses, we then additionally adjusted the underlying trial-generating model in the 3rd (and last) experiment. Again, we observed significantly smaller behavioral deviations from model predictions within run B of exp 3 compared to run B of exp 1. The deviation was comparable to that of exp 2. The total number of non-discounters further decreased on a descriptive level, although this was not confirmed statistically. In contrast to exp 2 (see Figure 3E), reaction times of exp 3 (see Figure 4E), however, were more in line with theoretical expectations by which reaction times close to the indifference point, that is, close to difficult choices, are slower compared with easy choices.

Interestingly, one of the most commonly applied models, the hyperbolic model, performed among the worst in predicting out-of-sample behavior (see also Supplementary Figure S6). With a correct prediction probability of on average ${\hat{p}}_{reward}$ = 0.57 and ${\hat{p}}_{loss}$ = 0.55 (evaluated across all runs and experiments, see Supplementary Figure S5 left), it performed only marginally above chance level. Overall, the hyperboloid models provided the highest prediction probability, averaged across experiments. The modified hyperboloid model was able to correctly predict behavior on average with 0.68 probability in the reward and 0.71 in the loss condition (see Supplementary Figure S5 left). It particularly excelled at predicting behavior in run A while staying close to the theoretical expectation in run B (see Supplementary Figure S5 right), as observed for several other models as well.

As most studies in the field do not report out-of-sample prediction errors (9, 21, 38, 41, 44, 49, 61, 63, 72, 74, 99–101), or report predicted log-likelihood (40), or predicted accuracies (54), which may be far above the predicted probabilities reported here, and since the prediction error depends on trial difficulty (i.e., on how close trials are to the indifference point and therefore on the precise experimental manipulation, cf. Section Behavioral Models and Model Parameters), the obtained values are difficult to compare. However, the results are in line with the few studies who have considered the modified hyperboloid model and have shown its superiority [(49, 61–63, 100); but see also (44)], and which show that the hyperbolic model is not a comparably good fit (61–63, 100).

The modified hyperboloid model is characterized by an additional free parameter *s* which scales the delay period in the discount factor (cf. Section Behavioral Models and Model Parameters) analogous to a psychophysiological power function [(9); see also (102)]. The power law, originating from psychophysics, describes the relationship between the intensity of a stimulus and the perceived magnitude increase in the sensation induced by the stimulus, which is modulated exponentially by a parameter, here *s* (102). In the present investigation, as often observed, *s* on average was smaller than 1 (38, 49, 61, 62), indicating a flattening of the discounting curve (cf. Figure 4G). This indicates that delay durations may not be perceived similarly, that is, objectively, across participants as indicated by e.g., the hyperbolic and exponential models, but there is additional inter-individual variability w.r.t. delay perceptions. This is in line with studies indicating that time perception plays a significant role in delay discounting [(103–107); see also (108)].

This scaling parameter *s*, as well as the discount parameter κ, moreover differed significantly between the reward and the loss condition. Both parameters were on average lower in the loss condition. Since the scaling parameter *s* was restricted between 0 and 1 [see also (109, 110)], smaller values here lead to a shrinking of the objectively experienced delay and thus to a lower degree of devaluation. Small κ values in the hyperboloid model similarly cause the discount factor to approach 1 such that effectively devaluation decreases. The fact that we found differences in both parameters, together with the fact that the hyperboloid model was superior to the hyperbolic at predicting the data, suggests that lower κ values alone were not sufficient to capture the weaker devaluation process observed in the loss condition. One may therefore speculate whether lower *s* values during loss discounting may be associated to a subjectively shorter perception of delays in this condition. Note though that this interpretation should be evaluated with caution since we observed a moderate correlation between *s* and κ in the reward condition. While a previous study evaluating the modified hyperboloid model did not find differences in the scaling parameter *s* between discounted rewards and losses (49), while others did not explicitly compare the parameters between tasks (38, 61, 62), the obtained results are in line with the frequent observation of lower discounting rates during loss discounting, also termed “sign-effect” [see also (41, 111); for an overview see (112)]. This sign-effect was also reflected in the lower frequency of discounted choices for the loss condition as compared to the reward condition observed for all runs and experiments despite explicitly prolonging delays for this condition (see Supplementary Results S2.1–S2.3).

Interestingly, the discount factor of the modified hyperboloid model was significantly related to subjective measurements: Subjectively reported impulsive behavior, as well as alcohol use behavior was negatively related to the discount factor, indicating that stronger temporal discounting was related to higher impulsivity and more alcohol use behavior. While this is in line with studies linking stronger discounting behavior to higher impulsivity as well as increased alcohol use behavior [(113–117); for an overview see (26, 118, 119)], other studies did not provide evidence for a direct link (120–122).

A crucial difference between our framework and other adaptive designs is that previous studies were mainly interested in the two-level comparison between hard and easy trials, i.e., trials close and far from the indifference point (31, 32, 43, 52, 54), or interested in choices around the indifference points [e.g., (31, 32, 43)]. By providing a formal condition for trial generation, our approach in contrast allows a more highly resolved and targeted grading of discounting probabilities. This includes the assessment of hard and easy trials, that is, trials with discounting probability 0.5 vs. discounting probability unequal to 0.5, as well as any other selected discounting probability on the probability measure (i.e., between 0 and 1). By inducing graded behavior, one presumably induces graded levels of cognition and associated neuronal responses. This facilitates the identification of brain regions or networks which co-vary with discounting probabilities, resolving the neural response at a finer scale and thus providing stronger evidence of the underlying neuronal mechanism (123–127). In addition, from a statistical point of view, generating more homogeneous experimental conditions across different levels of discounting behavior within subjects, should increase the statistical power needed to detect (differences in) the associated brain responses (128–130).

While most studies to date continue to focus on reward (rather than loss) discounting, we provide a general framework which is easily transferable to other scenarios. Although our approach did not work as well for the loss condition, that is, the average discounting frequency deviated somewhat from the model expectation, we did observe graded choice frequencies in response to the three experimentally manipulated levels for both reward and loss conditions. This (and even finer) gradation at the within subject level could be particularly helpful when studying the neurobiological underpinnings of a cognitive process, by providing a dimensional mapping from experimental trial to discounting probabilities.

Beyond that, many previous studies have focused on addressing the question of which discounting model best fits empirical data and how to adapt experimental trials to the individual. However, these studies mostly focused either on model comparisons [e.g., (38, 40, 49, 61, 72, 74, 89)], or on model-based trial adaptation [(31, 32, 36, 43, 54); but see (53)], but not on both. The latter is important though, since the success of model-based trial adaptation should naturally depend on the suitability of the model (see also Supplementary Figure S5). To our knowledge, only one study performed both model selection and design optimization simultaneously (53), selecting models on a subject-specific (as compared to group) level (which comes with its own advantages and disadvantages). However, this study as well as the other model comparison studies have mainly selected models based on in-sample error estimates [(9, 21, 38, 41, 44, 49, 61, 63, 72, 74, 99–101); but see also (40, 54)]. In-sample errors are susceptible to under-estimation of the PE due to e. g. overfitting, whereas out-of-sample errors represent more conservative and unbiased estimates (70, 88). They thus do not allow to quantify how well the models actually work at predicting unseen data (70). On the other hand, the studies focusing on adaptive designs have mainly focused on the hyperbolic model [e.g., hyperbolic only: (31, 32, 43, 54)], which performed particularly poorly in other studies [e.g., (53)], and yielded poor predictions as well as unreliable parameter estimates here.

One caveat of comparing multiple models as done in the present study is that it requires a sufficient number of trials. Other adaptive approaches which are often tuned to a single model have focused on optimizing efficiency and require a lower number of trials. A lower number of trials with equal reliability is desirable as it exerts less experimental burden on the participant. Overall, the applied number of trials varies highly between adaptive studies though, ranging from 5, ~10 and 98 trials in more recent approaches (31, 36) up to over 300 trials in more classical titration procedures [e.g., (32, 40, 43, 44, 47–50, 54, 89, 131, 132); with an average of around 95 trials (+/−77)]. The exact trial numbers may depend on subject specific parameters, and on how many delays and outcomes are applied. In an appealing Bayesian framework, Pooseh et al. (32) performed simulation analyses investigating the number of iterations necessary for parameter estimates to converge to their true values. Their results illustrate the dependency on the true parameter values and indicate that the classic amount adjusting method converges after 20–200 iterations (with high variance). Their Bayesian approach on the other hand starts to converge after around 50 trials for both κ and β evaluated on experimental data (32). Using adaptive design optimization [ADO; (36, 53)], recent studies demonstrated remarkable efficiency in measuring κ with high reliability in <10 trials, although the β parameter is inferred less reliably and likely requires more trials. Having now established a suitable model for our parametric method, one could perhaps improve the proposed framework by combining run A with one of these more efficient methods to further reduce the number of trials necessary in run A.

An interesting observation of the present study is that we noticed a high agreement in the discount factor of the hyperboloid model between both experimental runs and between reward and loss conditions. This agreement suggests that temporal discounting may be reliably measured (and may bear similarities in the processing of loss and reward) and is consistent with (the significantly) lower correlation of behavioral frequencies. In contrast, in the commonly used hyperbolic model, the relation between discount factors across runs (and partly across conditions), as well as the associations to discounting relevant measures such as impulsivity and alcohol use, vanished. On the one hand, this suggests that poorer behavioral models may provide more unreliable and biased parameter estimates, potentially also explaining difficulties in reproducibility between studies [see also e.g., (133) restricted reliability for hypothetical monetary outcomes; see also (36)]. On the other hand, it also shows that using appropriate behavioral models in combination with adaptive designs may even improve the valid and reliable measurement of cognitive function (superior to for instance behavioral frequencies). Especially considering the reproducibility crisis in psychological experiments [for overviews see (134–138)], such approaches could prove particularly beneficial [see also (139)].

Finally, we also address several limitations of the current study. First, our sample was highly dominated by women (with *N* = 145 women and *N* = 51 men). Although we found no differences in discounting behavior between women and men (cf. Supplementary Table S2), we cannot exclude that our findings generalize better to women. Second, although the proposed framework performed well within the reward condition, many non-discounters remained in the loss condition. It is unclear whether this may be attributed to yet suboptimal run A trial settings, inadequate to identify each participant's indifference point, or whether there is a true proportion of individuals in the population who do not exhibit loss discounting. The latter is not unlikely, as other studies with different settings have also found constant high rates of non-discounters (31, 33–35, 38, 40). However, it is also possible that the delays used in the current experiment were simply not long enough to tempt participants to discount future losses, masking the true proportion of non-discounters in the population. Future studies that explore the relationship of non-discounting to other subjective factors such as risk aversion, punishment sensitivity, reward sensitivity, preference uncertainty, and temporal uncertainty, or that systematically examine other trial settings, may help shed light on this question and reveal potential alternative discounting “styles.”

We also recognize that even in the reward condition, where choice frequencies on average matched well with model expectations, the behavioral variation was quite high. This could either be due to natural noise in the behavioral process, or that the true behavior generating model was not amongst the tested set. We cannot exclude that there is another model that describes the data better and would potentially further reduce the observed variation [see also 53]. For example, there is evidence that temporal discounting also depends on the tendency to avoid risks, often referred to as risk aversion (32, 40, 52, 73). Lopez-Guzman et al. (40) could for instance show that by inferring the individual's risk attitude on an independent task and adding it as an additional parameter to the discounting function, they could account for more behavioral variance in a temporal discounting task. It may also be reasonable to assume that individual participants are best described by different models (53), although inferring models on the single subject level limits comparability of associated neurobiological correlates.

## Conclusion

The present work proposes a model guided framework to evoke graded responses linked to cognitive function at a single subject level. Such a framework may be used in psychology, neuroscience, or psychiatry in the future to (a) measure cognitive function on a dimensional rather than dichotomous scale, (b) homogenize behavior across participants, (c) test the validity of a behavioral model, or (d) investigate the causal differences underlying heterogeneous behavior, which may benefit the investigation of cognitive mechanisms [see e.g., (140)]. Importantly, temporal discounting is a fundamental process underlying decision making and largely comparable between species (13). Given that similar decay functions of reward delay discounting have been observed in humans and rats (141), application of the here proposed adaptive experimental design to appropriate behavioral animal models may significantly enhance insights to the circuitry and molecular underpinnings of various neuropsychiatric disorders (142). Future studies are needed to assess whether our approach is suitable to dissolve discounting behavior on more than three levels, that is, on a more fine-grained dimensional spectrum of behavioral probabilities. We also propose a more general approach to create adaptive experimental designs based on the combination of behavioral models and model selection techniques. Our framework was tested in the context of temporal reward and loss discounting. It may however be generalized to other cognitive functions by using similar models which map actions probabilistically to an underlying cognitive process.

## Data Availability Statement

The datasets presented in this study, as well as the code needed to reproduce the findings presented in this study, can be found at https://github.com/GKoppe.

## Ethics Statement

The studies involving human participants were reviewed and approved by Medical Faculty Mannheim, University of Heidelberg (2019-633N). The participants provided their written informed consent to participate in this study.

## Author Contributions

GK, PK, and WHS conceptualized the study. GK, JT, MP, PH, PK, and WHS contributed to the design of the study. MP compiled the online experiment and collected the data. GK and JT performed the statistical analyses and wrote the manuscript. DD, GK, JT, MP, PH, PK, and WHS contributed to reading, revising, and approving the submitted manuscript. All authors contributed to the article and approved the submitted version.

## Funding

This work was funded by the German Research Foundation (DFG) within the collaborative Research Center TRR 265 subproject B08 granted to GK, PK and WHS.

## Conflict of Interest

The authors declare that the research was conducted in the absence of any commercial or financial relationships that could be construed as a potential conflict of interest.

## Publisher's Note

All claims expressed in this article are solely those of the authors and do not necessarily represent those of their affiliated organizations, or those of the publisher, the editors and the reviewers. Any product that may be evaluated in this article, or claim that may be made by its manufacturer, is not guaranteed or endorsed by the publisher.
