# Supplementary material for: A Model Guided Approach to Evoke Homogeneous Behavior During Temporal Reward and Loss Discounting
Source: Front Psychiatry. 2022 Jun 21;13:846119. doi: 10.3389/fpsyt.2022.846119 (PMC9253427; doi:10.3389/fpsyt.2022.846119)
Supplement: Supplementary file 1 [file Table_1.pdf]

Supplemental Material

Title: A model guided approach to evoke homogeneous behavior during temporal reward and loss discounting

Running title: Model-based induction of homogeneous behavior

Janine Thome[1,2*#], Mathieu Pinger[3#], Patrick Halli[3*], Daniel Durstewitz[1], Wolfgang H. Sommer[4*], Peter Kirsch[3,5], Georgia Koppe[1,2*]

[1]Department of Theoretical Neuroscience

[2]Department of Psychiatry and Psychotherapy

[3]Department of Clinical Psychology

[4]Institute for Psychopharmacology

Central Institute of Mental Health, Medical Faculty Mannheim, Heidelberg University, Mannheim, Germany

[5]Institute of Psychology, Heidelberg University, Heidelberg, Germany

# contributed equally

*** Correspondence:**

Janine Thome, janine.thome@zi-mannheim.de

Georgia Koppe, georgia.koppe@zi-mannheim.de

S1 Methods

S1.1 Generating trials by varying delays across subjects.

Eqn. (3) in the main manuscript may be rewritten as $V_2 = r_1 + \frac{\log(\frac{1-p_1}{p_1})}{\beta}$, where r_1 is the immediate outcome, p_1 is the (desired) immediate choice probability, and V_2 is the discounted value, implicitly containing the delay and the discount parameter. If we insert V_2, defined by the respective model, we may solve for the adaptive delay given a set of immediate and delayed outcomes, model parameters, and desired immediate choice probabilities. For instance, inserting V_2 of the hyperbolic model, yields $(\frac{1}{1+\kappa D})r_2 = r_1 + \frac{\log(\frac{1-p_1}{p_1})}{\beta}$, from which we can derive

$$D = \left(\left(\frac{r_1}{r_2} + \frac{\log(1-p_1)}{\beta r_2} - \frac{\log(p_1)}{\beta r_2}\right)^{-1} - 1\right)\left(\frac{1}{\kappa}\right),$$

as trial generating condition, where D is the delay, and κ and β are model parameters.

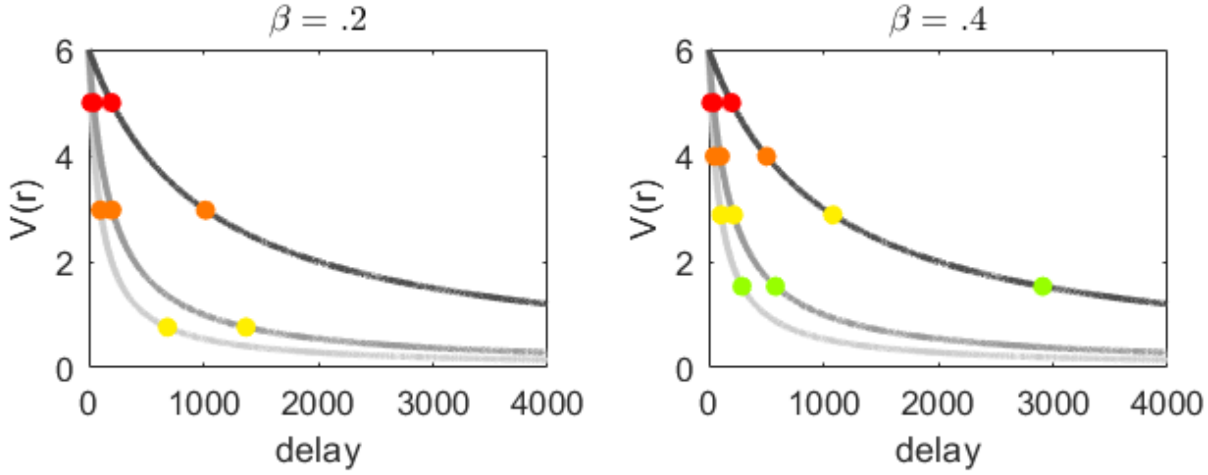

Fig S1. Illustration of method's operating principle when solving for delay rather than immediate outcome. In this example, the immediate reward was set to 5, and the delayed reward to 6. The 3 lines correspond to hypothetical κ values of .01 (light gray), .005 (gray), and .001 (dark grey). Colored dots mark the respective delays selected for each theoretical κ to obtain immediate choice probabilities of .5 (red), .6 (orange), .7 (yellow), and .8 (green). The left graph corresponds to subjects with β=.2 and the right to β=.4 (i.e., high sensitivity). To obtain similar discounting probabilities for subjects with different κ values (with same β), delays are selected such that the discounted value is equal across subjects (i.e., lies on a horizontal line). β tunes the difference between immediate and discounted outcomes, shifting the dots on the curves (i.e., discounted values) to the left. For larger β, shorter delays are necessary to discriminate between outcomes, consistent with higher sensitivity.

S2 Results

S2.1 Experiment 1

The frequency of discounted choices was lower in loss as compared to reward discounting in run A ($Z=6.07$, $p<0.001$), as well as run B ($Z=3.59$, $p<0.001$).

S2.2 Experiment 2

The frequency of discounted choices was lower in loss as compared to reward discounting in run A ($Z=5.89$, $p<0.001$), as well as run B ($Z=4.14$, $p<0.001$).

S2.3 Experiment 3

The frequency of discounted choices was lower in loss as compared to reward discounting in run A ($Z=4.99$, $p<0.001$), as well as run B ($Z=3.14$, $p=0.002$).

Table S1. Socio-demographic and subjective reports.

		Exp 1		Exp 2		Exp 3	
		N=98		N=50		N=50	
age (mean/SD)		32.44	11.55	31.38	9.79	31.86	9.24
gender (N)	female	65		37		43	
	male	32		12		7	
	diverse	1		1		0	
education (N)	primary	0		1		0	
	alevel	44		16		12	
	gcse	6		6		1	
	undergrad	32		15		25	
	grad	15		12		11	
	phd	1		0		1	
AUDIT-total		5.58	5.15	4.4	3.52	4.56	4.19
BIS-total		31.34	6.99	30.86	7.02	31.22	6.14
BIS-non-planning		11.29	3.19	10.6	3.41	10.9	2.84
BIS-motor		14	4.34	9.92	2.87	10.3	2.35
BIS-attentional		10.23	2.78	10.34	2.91	10.02	2.39

Legend. AUDIT = Alcohol Use Disorder Identification Test, BIS = Barratt Impulsiveness Scale; Exp = experiment; gcse = general certificates of secondary education

Table S2. Socio-demographic information across experiments with respect to gender (binary)

	**female**		**male**		**test-statistic**	
	N=145		N=51		Z	*p*
age (mean/SD)	31.49	12.09	33.94	9.89	1.09	0.275
**run A**						
% non-discounter	46.89		49.02			
% non-discounter reward	16.55		13.73			
% non-discounter loss	46.89		41.18			
reward imm choice freq (mean/SD)	37.21	25.21	32.98	26.09	0.88	0.381
loss imm choice freq (mean/SD)	80.53	16.94	84.72	22.83	0.39	0.693
reward explo-exploitan (mean/SD)	11.69	26.81	9.76	29.72	1.04	0.299
reward discounting par (median/perc)	0.02	[0.01,0.14]	0.01	[0.004,0.13]	0.87	0.382
reward scaling par (mean/SD)	0.57	0.38	0.60	0.37	0.49	0.618
loss explo-exploitan par (mean/SD)	27.36	42.04	28.49	42.9339	0.78	0.433
loss discounting par (median/perc)	0.002	[<0.001,0.03]	0.007	[<0.001,0.02]	0.79	0.424
loss scaling par (mean/SD)	0.52	0.35	0.53	0.36	0.19	0.845
**run B**						
% non-discounter	44.14		39.22			
% non-discounter reward	13.10		3.92			
% non-discounter loss	39.31		37.25			
reward imm choice freq (mean/SD)	42.96	24.53	50.04	27.25	1.74	0.081
loss imm choice freq (mean/SD)	71.92	30.19	68.76	28.94	0.72	0.469
reward explo-exploitan (mean/SD)	10.17	24.58	9.88	26.31	0.33	0.739
reward discounting par (median/perc)	0.01	[0.001,0.05]	0.02	[0.004,0.09]	0.28	0.781
reward scaling par (mean/SD)	0.65	0.37	0.65	0.36	0.01	0.995
loss explo-exploitan par (mean/SD)	33.68	43.65	30.75	44.87	0.19	0.844
loss discounting par (median/perc)	9.5x10-5	[<0.001,0.04]	0.004	[<0.001,0.04]	0.35	0.725
loss scaling par (mean/SD)	0.43	0.39	0.5	0.37	1.18	0.238

Legend. discount = discounting; explo = exploration; exploit = exploitation; imm = immediate; freq = frequency; par = parameter; SD = standard deviation; perc = percentile (25% - 75%); % = percentage

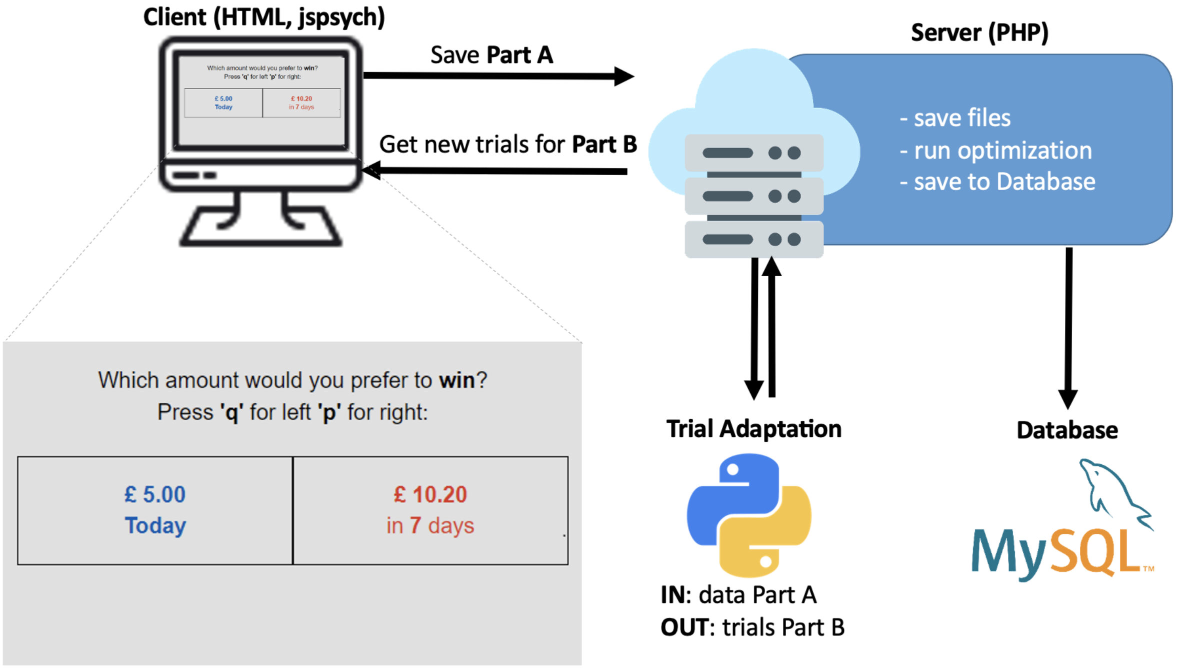

Fig. S2. Illustration of online paradigm technical information flow. Upper left: The reward and loss discounting paradigm was programmed in JavaScript using the open-source package 'jsPsych'. Lower left: Exemplary reward discounting trial prompting the participant to either press 'q' or 'p', if she/he wants to win £5 today (blue) or £10.20 in 7 days (red). Upper right: The experiment was hosted on a custom virtual server using Linux-Apache-PHP-MySQL. Lower right: Model inference on data from run A and trial generation for run B was realized on the custom virtual server using self-written Python scripts. Data was stored on the open-source data management system MySQL.

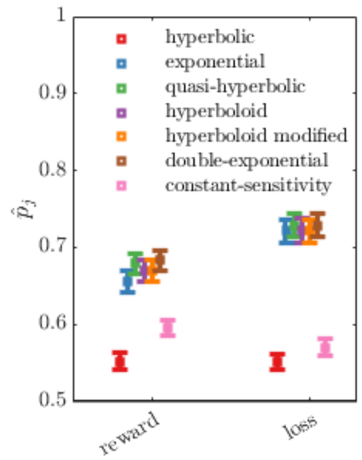

Fig. S3. Model comparison for experiment 1. Average predicted (out-of-sample) probability of observed responses $\hat{p}_j$ (y-axis) for reward and loss conditions (x-axis) for different models averaged over run A and B. Choice behavior of run B was predicted based on model parameters inferred on run A and vice versa. Choice behavior of the reward discounting condition was predicted by the hyperbolic model with $\hat{p}_{reward}$=.55, by the exponential model with $\hat{p}_{reward}$=.66, by the quasi-hyperbolic model with $\hat{p}$=.68, by the hyperboloid model with $\hat{p}_{reward}$=.67, by the modified hyperboloid model with $\hat{p}_{reward}$=.67, by the double-exponential model with $\hat{p}_{reward}$ =.68, and by the constant-sensitivity model with $\hat{p}_{reward}$=.60 (in order of the legend). Choice behavior of the loss discounting condition was predicted by the hyperbolic model with $\hat{p}_{loss}$=.55, by the exponential model with $\hat{p}_{loss}$=.72, by the quasi-hyperbolic model with $\hat{p}_{loss}$=.73, by the hyperboloid model with $\hat{p}_{loss}$=.72, by the modified hyperboloid model with $\hat{p}_{loss}$=.72, by the double-exponential model with $\hat{p}_{loss}$=.73, and by the constant-sensitivity model with $\hat{p}_{loss}$=.57.

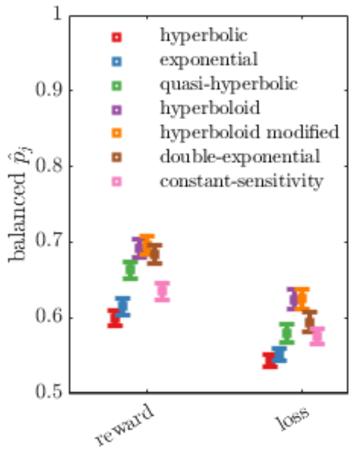

Fig. S4. Model comparison for experiment 2. Average predicted (out-of-sample) probability of observed responses $\hat{p}_j$ (y-axis) for reward and loss conditions (x-axis) for different models averaged over run A and B. Choice behavior of run B was predicted based on model parameters inferred on run A and vice versa. Choice behavior of the reward discounting condition was predicted by the hyperbolic model with $\hat{p}_{reward}$=.6, by the exponential model with $\hat{p}_{reward}$=.62, by the quasi-hyperbolic model with $\hat{p}$=.66, by the hyperboloid model with $\hat{p}_{reward}$=.69, by the modified hyperboloid model with $\hat{p}_{reward}$=.7, by the double-exponential model with $\hat{p}_{reward}$=.68, and by the constant-sensitivity model with $\hat{p}_{reward}$=.64 (in order of the legend). Choice behavior of the loss discounting condition was predicted by the hyperbolic model with $\hat{p}_{loss}$=.54, by the exponential model with $\hat{p}_{loss}$=.55, by the quasi-hyperbolic model with $\hat{p}_{loss}$=.58, by the hyperboloid model with $\hat{p}_{loss}$=.62, by the modified hyperboloid model with $\hat{p}_{loss}$=.63, by the double-exponential model with $\hat{p}_{loss}$=.59, and by the constant-sensitivity model with $\hat{p}_{loss}$=.58.

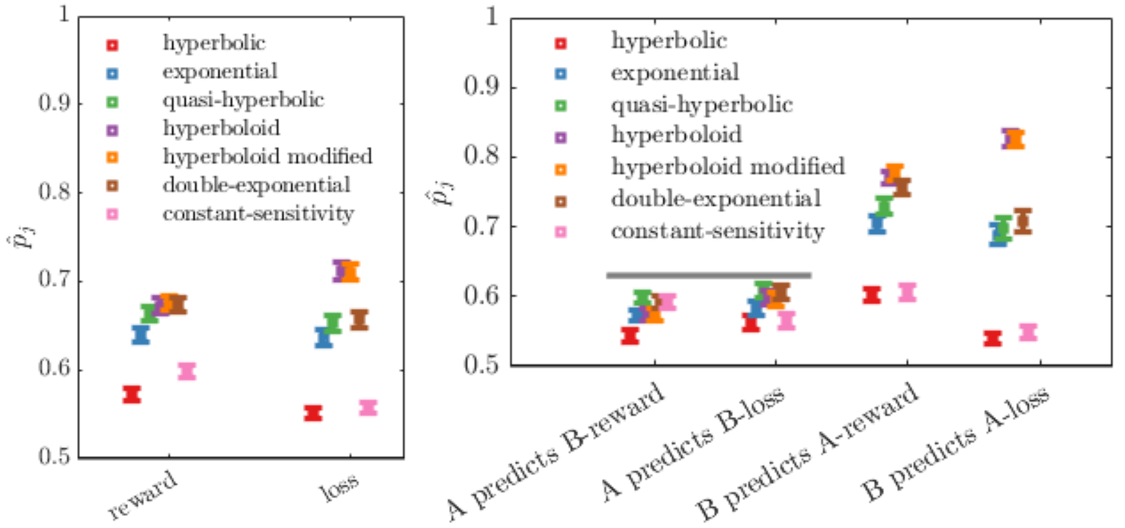

Fig. S5. Model comparison across all experiments. Left: Average predicted (out-of-sample) probability of observed responses $\hat{p}_j$ (y-axis) for reward and loss conditions (x-axis) for different models averaged over run A and B. Choice behavior of run B was predicted based on model parameters inferred on run A and vice versa. Choice behavior of the reward discounting condition was predicted by the hyperbolic model with $\hat{p}_{reward}$=.57, by the exponential model with $\hat{p}_{reward}$=.64, by the quasi-hyperbolic model with $\hat{p}$=.66, by the hyperboloid model with $\hat{p}_{reward}$=.67, by the modified hyperboloid model with $\hat{p}_{reward}$=.68, by the double-exponential model with $\hat{p}_{reward}$=.67, and by the constant-sensitivity model with $\hat{p}_{reward}$=.59 (in order of the legend). Choice behavior of the loss discounting condition was predicted by the hyperbolic model with $\hat{p}_{loss}$=.55, by the exponential model with $\hat{p}_{loss}$=.64, by the quasi-hyperbolic model with $\hat{p}_{loss}$=.65, by the hyperboloid model with $\hat{p}_{loss}$=.71, by the modified hyperboloid model with $\hat{p}_{loss}$=.71, by the double-exponential model with $\hat{p}_{loss}$=.66, and by the constant-sensitivity model with $\hat{p}_{loss}$=.56. Right: Same as left separated for predictions on run A and run B. When predicting run B based on models inferred on run A, all models perform below and close to the upper bound given by the theoretical expectation (horizontal grey line). When predicting behavior in run A based on models inferred on run B, the hyperboloid models show the highest prediction performance, while the common hyperbolic model performs particularly poorly.

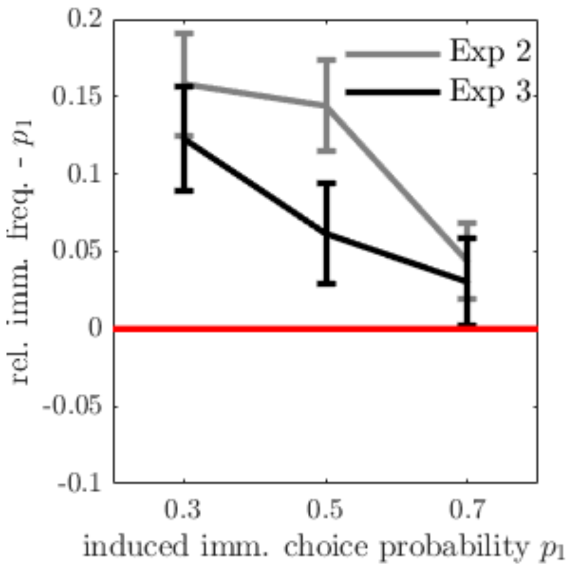

Fig S6. Investigation of model bias. The figure displays the deviation between observed relative immediate choice frequencies and induced immediate choice probabilities (y-axis), as a function of observed immediate choice probabilities (y-axis) in experiment 2 (grey) and experiment 3 (black), averaged across reward and loss conditions. The experiments differ w.r.t to whether choice probabilities were induced via the hyperbolic (experiment 2) or the modified hyperboloid (experiment 3) models. Descriptively, observed deviations are closer to 0 in experiment 3 indicating a lower bias in the induction of behavior for the modified hyperboloid model and thus indicating higher model validity. Statistically, we see a marginal difference within the .5 trail condition (p=.06).

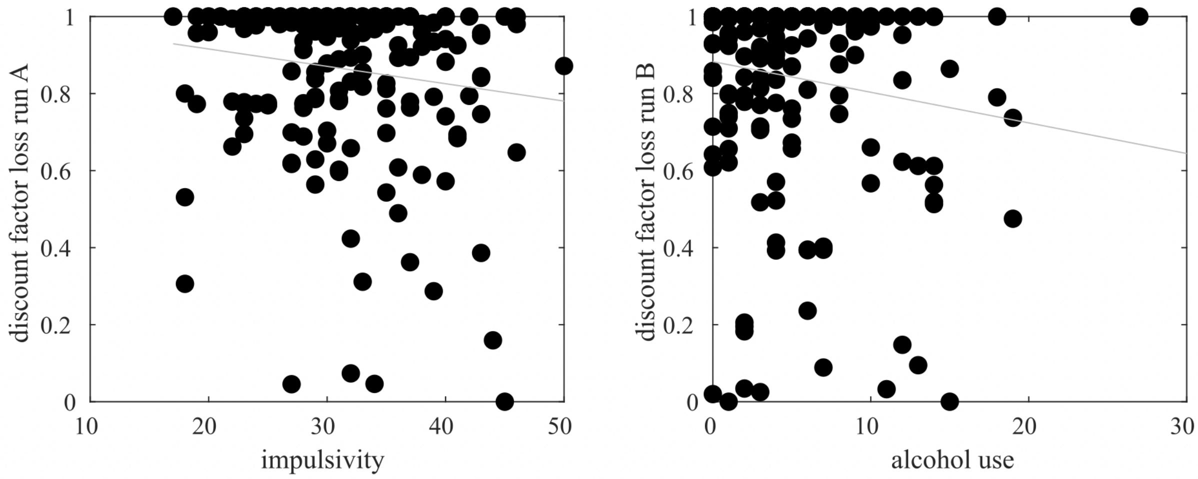

Fig S7. Correlation between subjective reports and discount factor of the modified hyperboloid model across all experiments. Left: Negative association between the discount factor (loss, run A) and impulsivity (BIS-total: r=-0.15, *p*=0.037). Right: Negative association between the discount factor (loss, run B) and alcohol use (AUDIT-total: r=-0.14, *p*=0.044).
